# Ecological Resilience of Restored Mediterranean‐Climate Woodlands to Experimental Fire

**DOI:** 10.1002/ece3.72445

**Published:** 2025-11-16

**Authors:** Ebony L. Cowan, Rachel J. Standish, Ben P. Miller, Russell G. Miller, Willa P. Veber, Joseph B. Fontaine

**Affiliations:** ^1^ School of Environmental and Conservation Sciences Murdoch University Murdoch Western Australia Australia; ^2^ Biodiversity and Conservation Science, Department of Biodiversity, Conservation and Attractions Kings Park Science Kings Park Western Australia Australia

**Keywords:** banksia woodland, disturbance, fire‐prone ecosystems, plant communities, resprouting, restoration, succession

## Abstract

The ability of restored sites to recover from subsequent disturbances is a key component of restoration success. Resilience is achieved when a restored site returns to its pre‐disturbance state, rather than shifts to a different one. In restored fire‐prone ecosystems, the drivers of post‐fire plant responses and resilience of plant assemblages to fire are underexplored. Exploration of these responses is used to predict and measure the resilience of restored ecosystems to disturbance, including whether the disturbance response was desirable or not. We implemented fine‐scale experimental fires in a post‐mining restoration chronosequence 14–27 years of age in Banksia woodlands, Western Australia. We sought to understand the effects of restoration age, fire impact, and soil conditions on post‐fire regeneration and survival of restored Banksia woodland plant assemblages. To assess early‐stage resilience to fire, we calculated four descriptors of ecosystem state: plant species density, species diversity, rarefied richness and functional redundancy, and compared how these changed following fire across the restoration ages and in comparison to nearby reference Banksia woodland. Ordinations and indicator species analyses were used to compare restored and reference sites. In restoration sites, restoration age, fire impact and soil conditions had little effect on plant regeneration and survival. Changes in diversity, rarefied richness and functional redundancy pre‐ to post‐fire in restored sites were typically similar to or less than that observed in reference sites. Broadly, our findings demonstrate the incomplete resilience of restored Banksia woodland to fire. Resprouters typically demonstrated poor resilience, through significant decreases in diversity and rarefied richness following fire in restored sites. They were under‐represented in restored Banksia woodlands, so further investigations into the establishment of resprouters in restored environments are required. Our findings also highlight the importance of utilising reference data and a broad range of descriptors to fully understand responses of restored plant assemblages to fire.

## Graphical abstract

Resilience of restored Banksia woodlands to fire is assessed using a multi‐faceted approach through investigating the drivers of post‐fire responses, and early‐stage reislience to fire. Findings differ among approaches, highlighing the need for a range of analyses to be used when assessing ecological resilience.

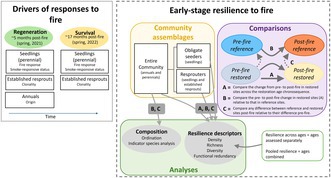

## 1 Introduction

Ecological restoration to improve the condition of degraded ecosystems is crucial for biodiversity conservation, with responses to disturbances being an important determinant of restoration success (Standish et al. 2014; Miller et al. 2017; Young et al. 2022; Cowan et al. 2021). Determining an ecosystem's ability to absorb changes in state variables and persist after a disturbance (i.e., ecological resilience; Holling 1973) is vital to understanding ecosystem dynamics and can help restoration practitioners determine successional trajectories. After a disturbance, resilient sites may be characterised by the maintenance of species, ecosystem structure and function (Walker et al. 2004).

Despite the importance of resilience for restoration projects, quantifying resilience is difficult, partly due to the vagueness of the term, varying responses of species to disturbance, and differentiating between desirable and undesirable changes to ecosystem states (Standish et al. 2014). This is particularly true in ecological restoration projects because resilience may develop over time, meaning that newly restored sites may revert to a degraded state after disturbance rather than following a desirable successional trajectory towards a reference state. Comparisons to reference sites are required to assess the restored site's responses to disturbance, if these responses are desirable, and if resilience is attained (Ross et al. 2004; Miller et al. 2017; McKenna et al. 2019; Harries et al. 2024).

Fire is a common disturbance globally that can substantially alter ecosystem development through changing species composition, ecosystem structure, and function (Keeley et al. 2011; Pausas and Keeley 2014, 2019). In fire‐prone environments, plant species have developed strategies to promote their regeneration and resilience following fire (Pausas and Keeley 2014). Key strategies include resprouting from surviving plant biomass (where species are referred to as resprouters; Clarke et al. 2013), or regeneration from fire‐responsive seedbanks following complete adult mortality (obligate seeders) with seeds often requiring exposure to heat or smoke for germination (Keeley et al. 2011). Development of these fire‐response mechanisms is typically shaped by fire intervals, where appropriate intervals allow sufficient development of seedbanks and resprouting capacity. However, seeding and resprouting may be compromised if fire intervals are shorter than the time to reproductive maturity, or longer than the lifespan of mature plants, seedbanks and resprouting organs (Clarke et al. 2013; Enright et al. 2014; Pausas and Keeley 2014). Initial, pre‐disturbance ecosystem states, and ecological (e.g., soil) and fire legacies (e.g., previous fire severity and coverage) also influence these responses and may be important for post‐fire resilience (Johnstone et al. 2016; Newman 2019).

Restoration projects may have their own set of unique complexities that may not be observed in comparable reference ecosystems, and vice versa, many of which are poorly understood. For example, in intact ecosystems, established resprouters are capable of resprouting shortly after disturbance, whereas restoration projects established from transferred topsoil are reliant on seedbanks until resprouting species have had time to develop rootstock and buds (Ross et al. 2004; Herath and Lamont 2009; Waryszak et al. 2021; Gerrits et al. 2023). Legacy effects of the restoration process such as soil compaction or altered soil profiles can limit respouters (Herath and Lamont 2009; Cowan, Fontaine, et al. 2023), influence soil seedbank composition (Cowan, Miller, et al. 2023), and alter rooting patterns (Carrick and Krüger 2007; Herath et al. 2009; Rokich 2016; Riviera et al. 2021; Figueiredo et al. 2023). In turn, these impacts may inhibit native plant responses to fire in restoration projects and instead favour the development of undesirable states such as the dominance of grassy weeds (Fisher et al. 2009).

Here, we implemented fine‐scale experimental fires to investigate early‐stage (~17 months post‐fire) resilience to fire in Banksia woodland plant assemblages restored after sand mining in southwestern Australia. This region has a Mediterranean‐type climate where plant traits adapted to fire are dominant reflecting the historical occurrence of fire in the landscape (He et al. 2016; Lamont and He 2017). Traits include resprouting, fire‐stimulated flowering, and fire‐cued seed germination (He et al. 2016; Lamont and He 2017). Understanding thresholds of resilience to fire disturbance can allow for the early identification of an undesirable development trajectory and the consideration of appropriate management strategies that may encourage restoration success (Cowan et al. 2021).

Using a chronosequence of restored sites aged between 14 and 27 years since the onset of restoration, we sought to determine the effect of restoration age on: (i) fire impact, which we expect to influence the (ii) drivers of post‐fire regeneration (measured ~5 months following fire) and survival (measured ~17 months following fire) for seedlings and resprouts, and (iii) the early‐stage resilience to fire. We expected restoration age to be a key driver of ecosystem development and responses to fire, and restoration age is commonly used when assessing restoration development and success (Cowan et al. 2021). As resilience is difficult to assess and interpret, we quantify early‐stage resilience to experimental fire through assessments of plant species density, Shannon‐Wiener species diversity, rarefied richness, and functional redundancy, and hereafter collectively refer to these as ‘resilience descriptors’. We also compare changes in resilience descriptors both pre‐ and post‐fire (after Holling 1973) and between restored and reference (i.e., intact native) Banksia woodland to infer resilience in the context of ecosystem development along a restoration trajectory towards a desired state. Finally, we utilise species composition analyses (ordination, indicator species analysis) to provide an additional assessment of resilience based on plant assemblages similarity between restored and reference sites.

## 2 Methods

### 2.1 Study Region

Banksia woodlands are a highly speciose (> 600 plant species) fire‐prone ecosystem with 2–3 co‐dominant *Banksia* tree species, *Allocasuarina fraseriana* 4–8 m tall with occasional emergent species of *Eucalyptus* 6–12 m tall, and contain a diverse sclerophyll understory (Stevens et al. 2016; Ritchie et al. 2021). They predominantly occur on the Swan Coastal Plain in the southwest of Western Australia, where minimum tolerable fire intervals are estimated at 8–10 years based on plant demography and fuel accumulation (Ritchie et al. 2021; Tangney et al. 2022; Miller et al. 2024, 2025). Post‐fire resprouting and obligate seeding are common traits among plant species (~78 and 22% of species respectively; Fontaine and Standish 2019), with many species able to both resprout and recruit from seeds following fire. Approximately 70% of species store their seeds in the soil (Rokich et al. 2001; Stevens et al. 2016) with seedbanks typically persistent between 1 and 40 years (Onans and Parsons 1980) and usually triggered for germination by smoke or heat (Bell et al. 1993; Turner et al. 2022). Canopy seed storage (serotiny) and release following fire are also present in species such as *Banksia*.

### 2.2 Study Site

This study was conducted at a sand mine operated by Hanson Heidelberg Construction Materials (hereafter ‘Hanson’), approximately 25 km northeast of Perth, Western Australia (lat. −31.76, lon. 115.95). The mine is in the Bassendean dune system (Bastian 1996) which has acidic, nutrient‐poor coarse sands with poor water‐holding capacity (Salama et al. 2005). The study site experiences a Mediterranean‐type climate. At the nearest climate station ~10 km northeast of the study site, maximum and minimum mid‐summer (January) temperatures had an average ± standard deviation of 33.5°C ± 1.7°C and 17°C ± 1.45°C respectively, with average maximum and minimum mid‐winter (July) temperatures of 18°C ± 0.9°C and 8.3°C ± 1.3°C between 1980 and 2020 (Bureau of Meteorology 2021). Mean annual precipitation was 634 ± 99.6 mm for the same period with majority of rainfall falling in May to August (Bureau of Meteorology 2021). The total rainfall for the period one year following the experimental burns in restored sites was ~769.2 mm.

Hanson has restored open‐cut sand mine pits to Banksia woodland most years since 1991. Before mining, vegetation is cleared, and the top 10 cm of topsoil that contains most of the soil seedbank is collected and stored for typically < 3 months. Due to relatively short topsoil storage times, the majority of the species present are able to recruit as seed viability is maintained (Stevens et al. 2016; Gerrits et al. 2023). Mining typically removes 5–50 m of the soil profile. After mining, sites have 2–4 m of sand or low organic content overburden sands returned, and 10 cm of the stockpiled topsoil deposited on top (B. Fruin & D. Hardy, pers. comm., November 2021; Stevens et al. 2016). To reduce compaction, sites are generally ripped to 10 cm depth after topsoil deposition (Table 1) which may influence future soil conditions and vegetation dynamics (Mounsey et al. 2021). Subsequent seeding and planting of difficult‐to‐establish species, including resprouters and serotinous species, often occur within 4 years of topsoil deposition. Typically, germination of smoke‐responsive species occurs within the first year after topsoil application, potentially due to the release of the same active ‘smoke’ chemical during topsoil transfer and ripping (Merritt et al. 2006; Daws et al. 2014; Fowler et al. 2015).

**TABLE 1:** Age, soil conditions and restoration practices of restored Banksia woodland sites surveyed.

Restoration age (years)	Year established	Soil compaction (MPa)		Soil field capacity (%)	Ripping depth	Soil profile composition
		10 cm	30 cm			
14	2007	0.64–0.88	1.28–1.48	14–26	Shallow	Sand
16	2005	0.7–1	1–2.16	19–24	None	Clay
17	2004	0.76–1.16	1–1.48	19–26	Shallow	Sand
18	2003	0.64–0.82	0.84–1.48	20–24	None	Sand
22	1999	0.9–1.18	1.18–2.26	9–23	Shallow	Sand
24	1997	0.68–0.88	0.78–1.14	11–25	Deep	Overburden
27	1994	0.86–1.44	2.4–4.4	9–20	None	Overburden

*Note:* Soil compaction data is the range of averages per plot, while field capacity is the range of plot values (*n* = 5). Ripping depth: shallow = 10–15 cm, deep = 50 cm, MPa = megapascals.

### 2.3 Restored Banksia Woodland Data Collection

In October—November 2019 (spring), we measured plant species composition at seven sites with restoration ages between 14 and 27 years dating from the placement of returned topsoil (Table 1). We established five 4 × 4 m (16 m^2^) plots per restoration age, with plots randomly placed throughout each restored site at least 20 m apart. In each 4 × 4 m plot, we counted the number of established individuals greater than 2–3 years old for all perennial species. In two 0.7 × 0.7 m subplots nested in the southwest and northeast corners of each 4 × 4 m plot, we also counted all individuals of all species, including annuals and seedlings of perennial species.

**FIGURE 1:**
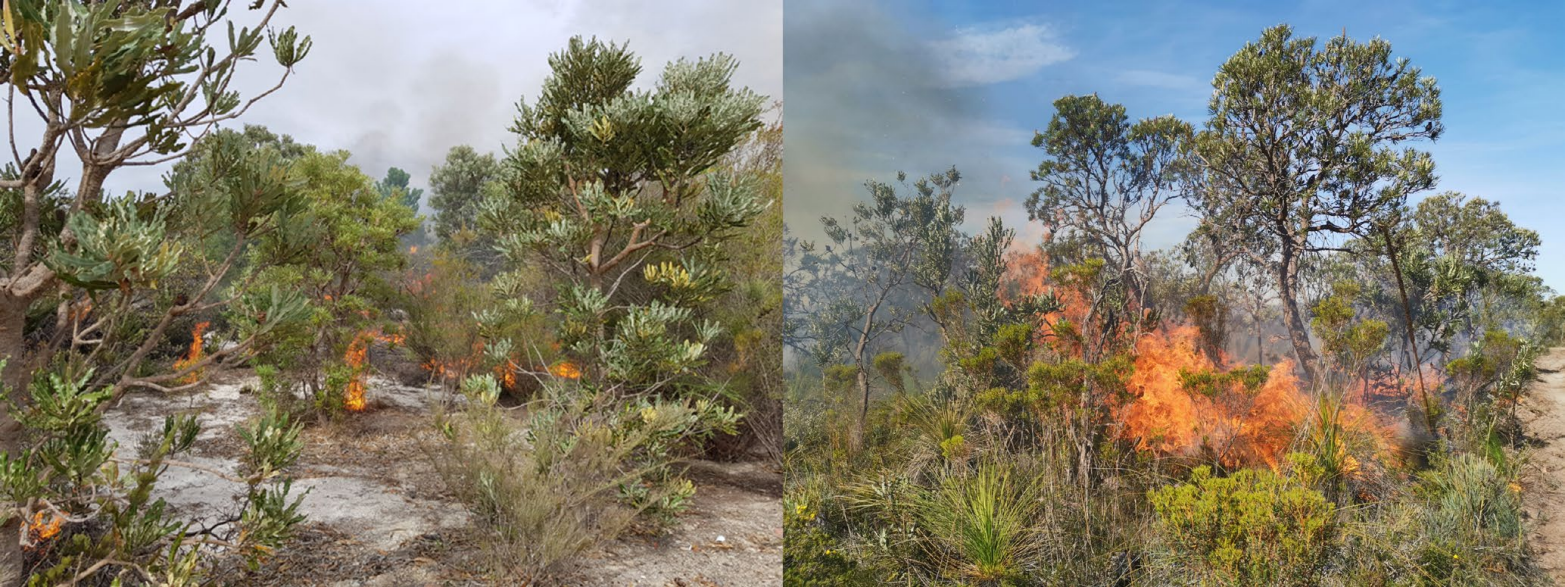
Fire in restored (left) and reference (right) Banksia woodland. Photos provided by E.L.C. and J.B.F.

In May 2021 (autumn), the restoration sites were burnt in experimental prescribed burns by the Western Australian Department of Biodiversity, Conservation and Attractions (DBCA; Figure 1). Where fire spread within sites was impeded due to low fuel connectivity, fire was applied directly to plants and combustible litter in plots using drip torches. Fire was not attempted in sites < 13 years old given that these ages had high bare ground cover and plots were unlikely to burn, even with direct ignition (DBCA, pers. comm., June 2019; Appendix S1: Figure S1).

Fire impact was assessed within three days of the burns. To do so, we estimated fire coverage as the percentage area of each subplot burnt which was largely based on the presence of burnt litter, and fire severity as the proportion of plants scorched within each 4 × 4 m plot. For trees, the estimated percent of crown burnt and the height of charring on stems were also recorded.

In October 2021 (spring; ~5 months following fire) we assessed initial post‐fire regeneration in the burnt plots. Mature perennial plants in the 4 × 4 m plots were again counted by species and recorded as either survivors, those not impacted by fire and still alive; or established resprouts, individuals which had been burnt by fire and subsequently resprouted. In the 0.7 × 0.7 m subplots, all individuals were counted and classed as follows: seedlings, individuals that had germinated within the first 5 months following fire, survivors or established resprouts (defined as above). Due to low numbers of smoke and/or heat‐responsive species seedlings in some plots, particularly plots with little fire coverage, additional 0.7 × 0.7 m subplots were added in the other corners (southeast and northwest) of the 4 × 4 m plots until at least 50 perennial seedlings were recorded in total for each plot. Twelve (34%) 4 × 4 m plots had two subplots, 20 (57%) had four subplots and three (9%) had eight subplots. In October 2022 (spring) ~17 months post‐fire, we assessed the survival of seedlings and resprouts by re‐counting individuals in the burnt plots using the same methods.

### 2.4 Reference Banksia Woodland Data Collection

Data in reference (i.e., intact, native) Banksia woodland were collected from sites 1.3 to 8.5 km from the Hanson mine (Appendix S1: Table S1). Two sites aged between 10 and 22 years since the last fire were measured one year following the fire (hereafter post‐fire reference sites), with nine sites measured at varying intervals from 4 to 49 years since the fire (hereafter pre‐fire reference sites). Each site had 5–7 replicate 4 × 4 m plots. All perennials were measured in spring using the same methods as above. In the two sites monitored one year following the fire, plants were recorded as survivors or established resprouts as per above, with seedling regeneration assessed in two replicate subplots, the same size as those used in restored plant data collection (0.7 × 0.7 m). Fires in reference Banksia woodland plots were conducted as part of DBCA's prescribed burning program. Data were collected as part of a broader project investigating fire interval effects in intact Banksia woodland.

### 2.5 Explanatory Environmental Variables in Restored Sites

To determine the effect of soil conditions on seedling establishment and resprouter vegetation regrowth, we assessed soil compaction in dry soils in November 2021 (late spring). For each restoration age, we measured 25 replicates of soil compaction using an Eijkelkamp Penetrologger 6.08. We did not expect field capacity, defined as water holding capacity after excess water has drained, to be as variable as compaction due to soil profiles being established in a similar manner within each restoration age (Table 1). Therefore, we collected one replicate per plot to a depth of 6 cm in the soil profile to measure field capacity. In the laboratory, soils were wet until saturation occurred, drained for 24 h and oven‐dried at 80°C until constant weight was achieved.

## 3 Data Processing

**FIGURE 2:**
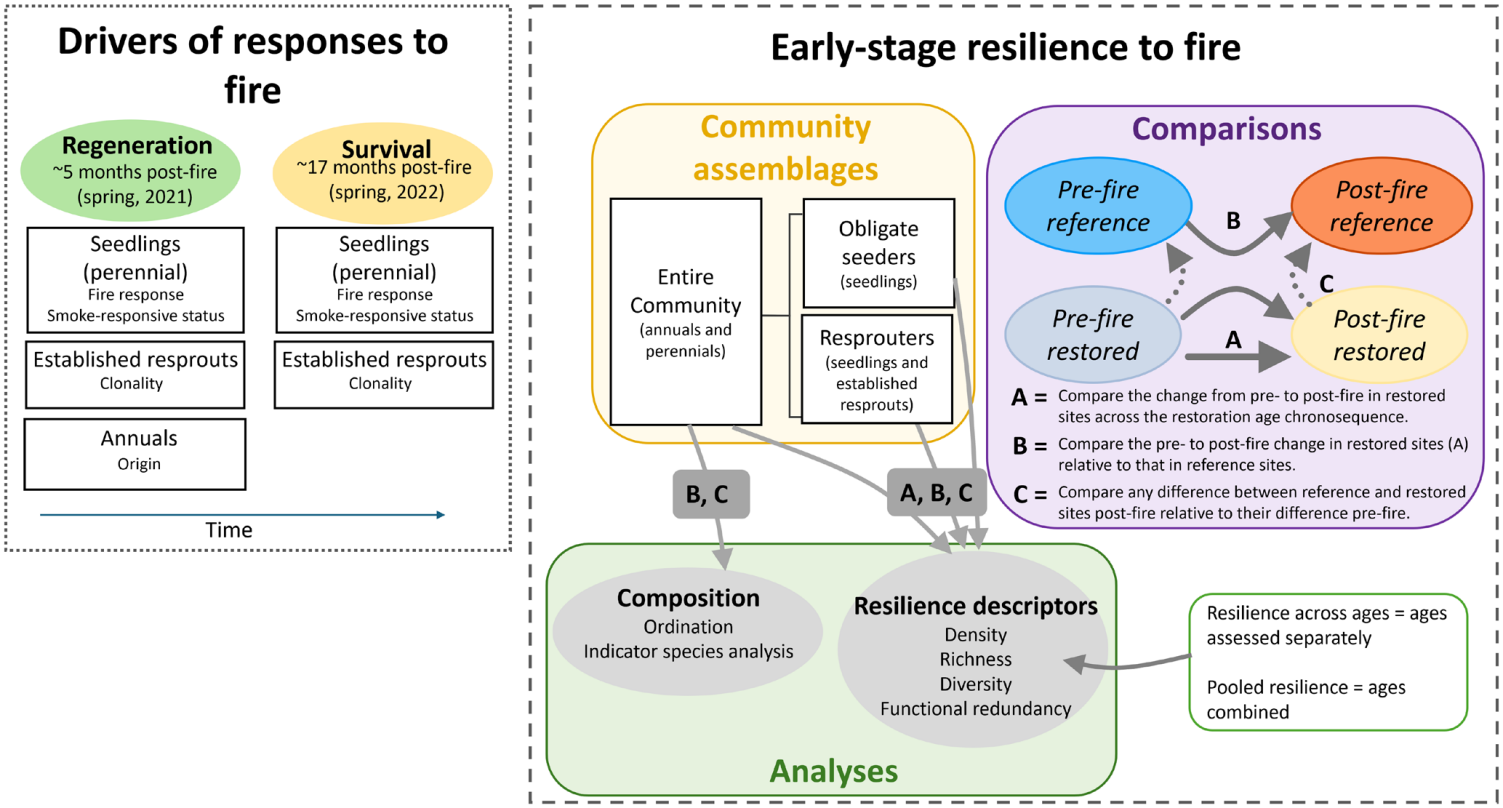
Diagram summarising plant trait types and assemblages responses considered in this study, where ovals refer to response variables while rectangles with a white background represent plant assemblage groups.

We assessed drivers of (i) post‐fire responses on regeneration at 5 months and survival at 17 months and (ii) early‐stage resilience to fire using community‐level data and a comparison to reference Banksia woodland (Figure 2).

Expecting drivers of post‐fire responses (Figure 2) to vary between seeders and resprouters, we assessed the seedling recruitment of annual and perennial seeders, and the resprouting of pre‐fire established individuals (hereafter ‘established resprouts’; Figure 2). Survival of ~5‐month post‐fire individuals to ~17 months was assessed for perennial seedlings and established resprouts, but not annuals as individuals do not survive from one year to the next. Separation of plant assemblages was based on species‐level traits that are expected to have a strong influence on fire response: seedlings were separated by the smoke‐responsive status of their seedbank and their fire‐response type (obligate seeder vs. resprouter), while established resprouts were separated based on clonal vs. non‐clonal growth habit (Text S1; Figure 2). Data on plant traits were collated from relevant databases (Appendix S1: Table S2) and supplemented with field observations (E. Cowan, pers. obs., 2021–2022). We separated germinants according to their expected germination response to smoke to understand seedbank dynamics and the resilience of species not previously assessed at Hanson (Cowan, Miller, et al. 2023), allowing for a more complete understanding of resilience to fire.

To assess early‐stage resilience to fire, we compared the ~17‐month post‐fire restored sites to pre‐fire Banksia woodland in both restored and reference sites (Figure 2). Early‐stage resilience to fire was assessed with the ~17 month post‐fire data as plants were established and reflected the successional trajectory. Because plants (particularly obligate seeders) senesce over time, density and richness typically decline with increasing fire intervals (Hobbs and Atkins 1990; Enright et al. 2014) or time since the onset of restoration (Standish et al. 2021, 2023). Therefore, we separated our reference sites into groups based on their fire intervals for comparison with pre‐ and post‐fire restored sites. For post‐fire reference sites, we used two sites for analysis which were burnt at different fire intervals (10 vs. 22 years since last fire; Appendix S1: Table S1). Due to substantial differences in density (Appendix S1: Figure S2a) we assessed these sites separately. For pre‐fire reference sites, we grouped sites into three age classes: 4 years post‐fire (two sites), 10–24 years since fire (six sites) and 49 years since fire (one site) as density and richness were similar among these age groups (Appendix S1: Table S1; Figure S2). Among the restored vs. reference, pre‐ vs. post‐fire ecosystem types, we separated the plant community into three assemblages based on mode of regeneration: (1) the entire community (all annuals and native perennials), (2) obligate seeders (perennial) and (3) resprouters (perennial; Figure 2). Annuals were not assessed on their own for the reasons mentioned earlier.

## 4 Data Analysis

For all analyses, we removed ephemeral species (i.e., Orchidaceae and *Drosera* species; making up < 2% of counts) as variation in yearly weather patterns influenced the timing of their aboveground presence. We also removed invasive perennials due to their low abundance across the study (< 6% of counts). Plants unaffected by fire (individuals not burnt) were removed from the dataset. Analyses were conducted in R Studio (R Core Team 2020) with data visualisations created using *ggplot2* (Wickham 2016), *cowplot* (Wilke 2020), and *ggnewscale* (Campitelli 2022). Here and throughout, we interpreted a lack of overlap in 95% confidence intervals (CI) as evidence of a statistical difference at *p* < 0.05 (Ramsey and Schafer 2002).

### 4.1 Quantifying Drivers of Post‐Fire Seeding and Resprouting Responses in Restored Banksia Woodland

To assess post‐fire regeneration and survival in restored Banksia woodland, we first modelled different plant assemblage groups of restored Banksia woodland against restoration age, fire impact, and soil variables (Figure 2). However, due to similar responses across the restoration chronosequence and little impact of plant traits, fire impact, and soil conditions, we pooled species counts per plot and restoration age to provide an assessment of post‐fire seedling and resprouting responses. See Text S2 for detailed modelling procedures.

### 4.2 Assessments of Early‐Stage Resilience to Fire

We assessed early‐stage resilience of restored Banksia woodland plant assemblages to fire using three approaches: (1) resilience across restoration ages (restoration age analysed individually), (2) pooled resilience (restoration ages combined), and (3) changes in composition pre‐ and post‐fire, and compared with reference sites (Figure 2). Approaches (1) and (2) quantified resilience using ‘resilience descriptors’, while changes in composition were assessed using ordinations and indicator species analyses (Figure 2). All these descriptors are commonly used to quantify resilience and changes following disturbances (Cowan et al. 2021).

#### 4.2.1 Resilience Across Restoration Ages

We expected restoration age to influence early‐stage resilience to fire due to reproductive or resprouting capacity potentially developing with time. Analysis of seedbank development (Cowan, Miller, et al. 2023) and resprouter responses to fire (Cowan, Fontaine, et al. 2023) at Hanson has revealed mixed importance of restoration age, and we sought to determine its effect on the responses of the entire plant community to fire. Therefore, for our first assessment of resilience, we assessed the impact of fire on ecosystem states through assessments of pre‐ and post‐fire states per restoration age (hereafter ‘resilience across ages’; Figure 2). For each restoration age, we compared whether restored sites were similar to reference sites by calculating means and 95% CIs of the previously mentioned assemblages and reference site groups. For the reference site summaries, ages were combined within pre‐ and post‐fire groupings due to little difference in the values of most descriptors.

#### 4.2.2 Pooled Resilience

To provide a broader overview of restored sites' resilience to fire, means and 95% CIs for restored pre‐ and post‐fire, and reference pre‐ and post‐fire states across all restoration ages (hereafter ‘pooled resilience’) were calculated. Using these data, we assessed the resilience of the restored sites in comparison to reference sites (Figure 2). This approach permitted the quantification of change post‐fire in restored sites versus that of reference sites. These comparisons were based on the amount and direction (positive vs. negative) of change in states of resilience descriptors, and asked: (A) how did restored sites change following fire (compared restored pre‐ vs. post‐fire states), (B) is resilience in restored sites similar to that of reference sites (i.e., has restored sites' resilience to fire been restored assessed through comparing change from fire in restored vs. reference), and (C) are restored sites on a trajectory towards reference states following fire, which was determined through comparisons of any differences in post‐fire restored and reference states, versus pre‐fire restored and reference states (Figure 2).

#### 4.2.3 Resilience Descriptors

We assessed both resilience across restoration ages and pooled resilience using density per species, Shannon Wiener diversity, rarefied richness, and functional redundancy of the plant assemblage (Figure 2). Density per species, Shannon Wiener diversity, and rarefied richness are commonly used to assess resilience and responses to disturbances in both restored (Cowan et al. 2021) and intact ecosystems (Albrich et al. 2020; Nikinmaa et al. 2020), while functional redundancy provides an alternative assessment of resilience that incorporates species traits (Walker 1992; Cowan et al. 2021; Standish et al. 2014).

Variables were calculated for each assemblage (Figure 2). Density measures were assessed within smoke‐response and unlikely smoke‐responsive classes to investigate possible different responses and assess their development of resilience, but diversity, rarefied richness and functional redundancy were not, as some ages had low replication of species in trait groups, preventing the calculation of descriptors. Shannon Wiener diversity is based on the number of species and relative abundances, while rarefied richness provides an indication of expected richness based on sample sizes (Hurlbert 1971; Oksanen et al. 2020). Rarefied richness was calculated to account for varying sample sizes between pre‐ and post‐fire and restoration and reference sites. Both diversity and rarefied richness were calculated in the R package *vegan* using the functions “diversity” and “rarefy” respectively (Oksanen et al. 2020).

Functional redundancy measures the level of redundancy in an ecosystem based on species counts and species trait values (Walker 1992; Díaz and Cabido 2001) and may be a proxy measure of resilience (Standish et al. 2014; Cowan et al. 2021). Theory suggests that communities with more redundant species (i.e., more species occupying a similar functional role to each other) should be buffered against the loss of ecosystem functioning and future resilience even if individual species are lost (Díaz and Cabido 2001; Oliver et al. 2015; Biggs et al. 2020). We calculated functional redundancy using a variety of plant traits that may influence assemblage responses to fire, including: fire‐response, longevity, species origin, growth form, smoke‐responsive status, seed storage location, microbial associations, seed mass and specific leaf area (Appendix S1: Table S3). Functional redundancy was calculated by first determining species similarity in trait space using Gower distances in the *FD* package (Laliberté and Legendre 2010, 2014). Gower distances can tolerate missing trait data (Brown et al. 2012). Next, using trait similarity and species density, in the *adiv* package (Pavoine 2020), we calculated functional redundancy using the “uniqueness” function. Removal of traits where a high proportion of species had missing trait data had minimal influence on functional redundancy values (Appendix S1: Figure S3), so we kept all traits in the analyses.

### 4.3 Assessments of Resilience Based on Assemblage Composition

In restoration projects, a common challenge is restoring a similar community in terms of the number of species and their abundance to that in reference ecosystems (Herath et al. 2009; Mounsey et al. 2021; Riviera et al. 2021). Therefore, an important component of resilience in restored ecosystems is determining the similarity between restored and reference sites (Cowan et al. 2021). Using data on the entire plant community in restored and reference sites across pre‐ and post‐fire states, we conducted an indicator species analysis (ISA), and non‐metric multidimensional scaling ordination (NMDS; Figure 2). We separated sites by age to determine potential successional trajectories in restored sites and their changes with fire, and how this compares to reference ecosystems.

An ISA was conducted to understand how species trait dominance contributes to resilience and change between sites and their age. Species with high indicator values suggest high frequency and abundance within a given age (De Cáceres and Legendre 2009). We combined indicator species by trait type within ecosystem type (restored vs. reference) and site age for ease of visualisation due to the high number of indicator species. ISA was conducted in the R package *Indicspecies* (De Cáceres and Legendre 2009). We report indicator species where *p* < 0.05.

To assess ecological resilience based on the return of sites to their pre‐disturbance state or a reference state, we conducted a 3D NMDS using the Hellinger transformed Bray–Curtis similarity of pre‐fire and ~17 month post‐fire restoration, and reference (pre‐ and post‐fire) Banksia woodland. The Hellinger transformation divides all values in a row (site) by the row sum, and then square root transforms these values, and is appropriate for datasets with many zeros, such as the dataset used here (Legendre and Gallagher 2001; Legendre and Legendre 2012). NMDS was conducted for the entire community (annuals and native perennials) in the R package *vegan* (Oksanen et al. 2020). We added polygons for each ecosystem type (restoration vs. reference, pre‐ vs. post‐fire), and centroids for each age for ease of visualisation. We also connected pre‐fire to post‐fire restoration age centroids using arrows to show how these assemblages have shifted following fire. Due to density differing between plant trait types and ecosystem types, and the potential impact on similarity in composition, we added a biplot of the proportion of individuals per plot for the previously defined plant trait classes (i.e., native obligate seeder, native resprouter, native annual, and invasive annual) using the “envfit” function (Oksanen et al. 2020). We used analysis of similarity between ages and ecosystem type in *vegan* (Oksanen et al. 2020) to determine if groups were different.

## 5 Results

### 5.1 Characterising Banksia Woodland

Across the entire restoration chronosequence, we found 113 species including 5 invasive perennial species. Of these 108 species (excluding invasive perennials), 49 were resprouters, 26 obligate seeders, 19 native annuals, and 14 invasive annuals. Of the native perennial species, 19 (73%) obligate seeders and 36 (73%) resprouters were smoke‐responsive (Appendix S2: Table S1). For species capable of resprouting, there were 24 clonal and 25 non‐clonal species respectively in restored sites. Twenty‐seven species found pre‐fire were not found post‐fire and 63% of these were resprouters, with 16 species found in post‐fire only (63% of these were native annuals; Appendix S2: Table S1).

Of the 108 species found in restoration sites (excluding the 5 invasive perennial species), 97 (90%) were also found in reference Banksia woodland (Appendix S2: Table S1). There were an additional 65 species found in reference sites only, with the majority of these being perennial species (37 perennial resprouters, 15 perennial obligate seeders; Appendix S2: Table S1).

### 5.2 Fire Impact in Restored Banksia Woodlands

**FIGURE 3:**
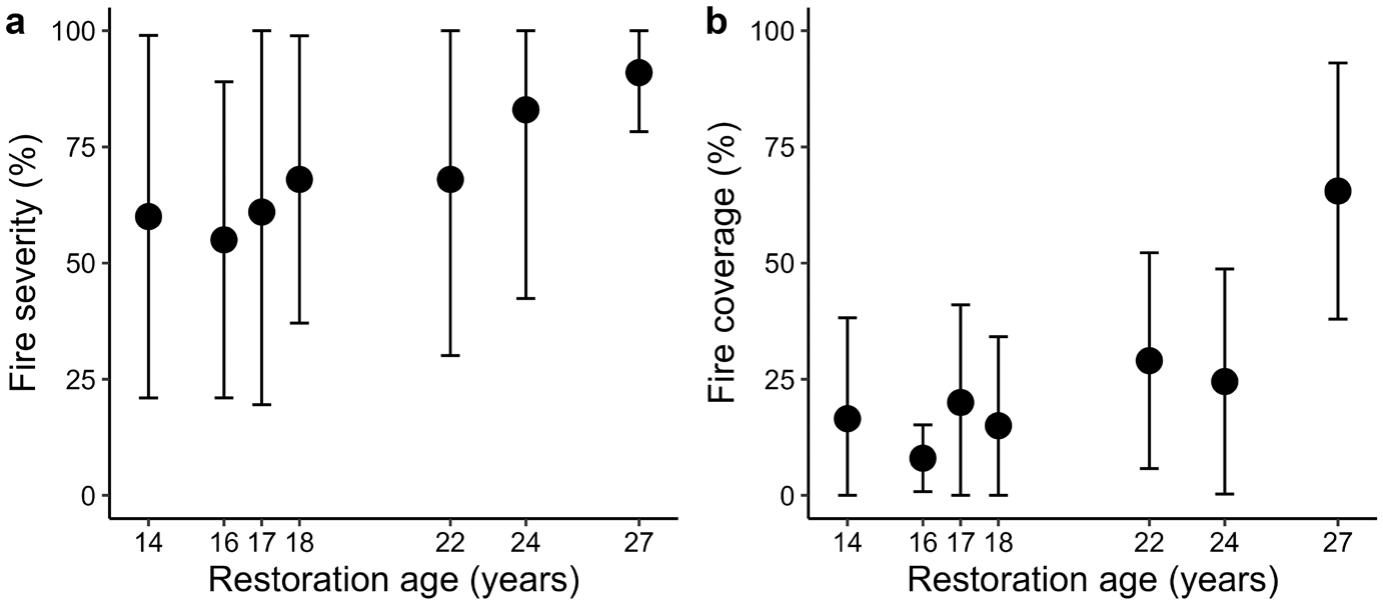
Mean±95% CI of percentage of fire (a) severity and (b) coverage across the restoration chronosequence.

Fire severity (percentage of shrubs scorched) in restoration plots burnt > 14 years was highly variable (55%–91%) and had no trend with time due to overlapping 95% confidence intervals (Figure 3a). Similarly, fire coverage (i.e., the amount of each subplot burnt) was highest in the oldest site (65% in age 27 vs. 5%–30% in ages 14–24) but not significantly higher due to overlapping 95% confidence intervals (Figure 3b).

Experimental burns had little impact on tree canopies as 85% of plots had > 90% unburnt tree canopies, and no plots had > 50% tree canopy unburnt. Forty percent of plots had charring on tree stems. Twenty‐five percent of plots had char heights < 25% of the total tree height on at least one tree, with the char heights typically < 20 cm high. One plot had charring at 50% of the height of one tree.

### 5.3 Post‐Fire Regeneration and Survival

**FIGURE 4:**
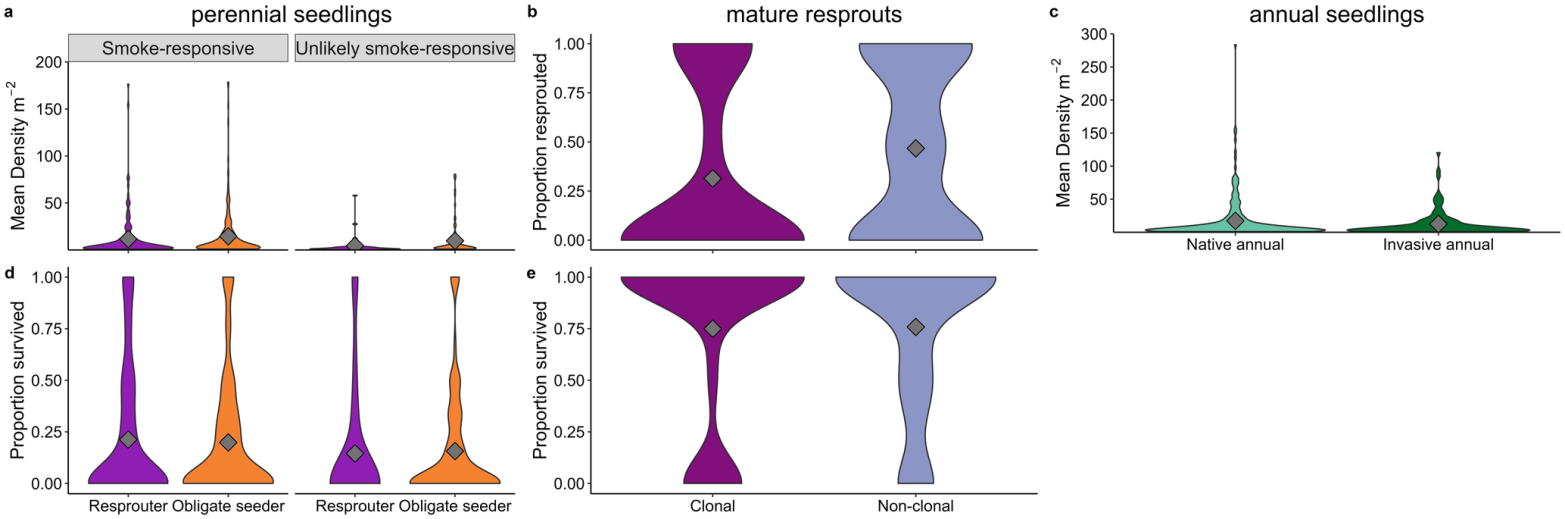
Violin plots for regeneration at ~5 months post‐fire of (a) perennial seedlings, (b) established resprouts (proportion of pre‐fire individuals that had resprouted at ~5 months following fire) and (c) annual seedlings, and ~17 month survival proportions (of ~5 month data) of (d) perennial seedlings and (e) established resprouts. Data are species counts (a, c) or proportion (b, d, e) per plot and restoration age combination. Grey diamond = mean. *n* = 31–243.

At ~5 months post‐fire, there was evidence of recruitment from the seedbank for both perennial and annual species with substantial seedling density across most restoration ages (Appendix S2; Figure S1a–c). When pooled across restoration ages, the density of smoke‐responsive perennials was more variable than that of unlikely smoke‐responsive species (Figure 4a). Across the restoration chronosequence, restoration age, native vs. invasive origin (assessed for annuals only), fire‐response type (assessed for perennial seedlings only), and fire coverage had no clear effect on seedling density (Appendix S2: Figure S1a–c; Table S2).

Following fire, persistence via resprouting occurred across the restoration chronosequence. The proportion of pre‐fire established resprouters (those capable of resprouting) that had resprouted and survived fire varied widely (Appendix S2: Figure S1d). Mean resprouting proportions were similar between non‐clonal (0.47) and clonal (0.31) resprouters (Figure 4b), with resprouting proportions also similar across most restoration ages (Appendix S2: Figure S1d). Restoration age and fire severity (the proportions of plants scorched) did not have a significant effect on the proportion of plants that resprouted, nor did interactions between age or severity with clonality (Appendix S2: Table S2).

The proportion of seedlings and resprouts of perennial species present at ~5 months that survived to ~17 months was variable across the restoration chronosequence (Appendix S2: Figure S2). The average survival of perennial seedlings was similar regardless of a species' smoke‐responsive status or fire‐response type (mean proportions = 0.15–0.20; Figure 4d). For established resprouters, survival from ~5 to ~17 months was relatively high and did not differ among clonal and non‐clonal species types (proportions = 0.75 vs. 0.76; Figure 4e; Appendix S2: Figure S2c). When separated by restoration age, both perennial seedlings and established resprouts' survival was not influenced by restoration age, soil compaction, or field capacity (Appendix S2: Table S3). On average, 43% of pre‐fire resprouters were alive at ~17 months post‐fire.

### 5.4 Resilience Across Restoration Ages

**FIGURE 5:**
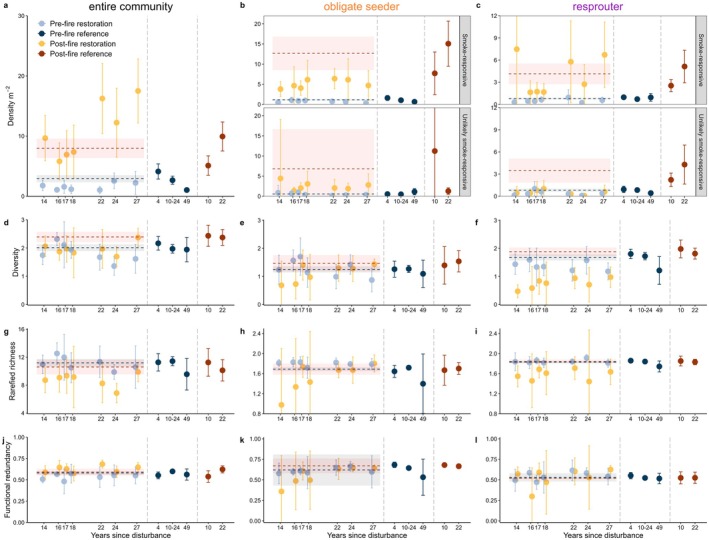
Mean±95% CI values for entire community (annuals and native perennials) and perennial obligate seeder and resprouter density, diversity, rarefied richness and functional redundancy against years since disturbance in Banksia woodlands. Years since disturbance refers to restoration age in restoration sites and years since fire in reference sites. Colours reflect ecosystem types and state (pre‐ vs. post‐fire). Bands and dotted lines over restoration data refer to means (dotted line) and 95% CI of post‐fire (red) and pre‐fire (blue) reference Banksia woodlands data. Post‐fire restoration data was measured ~17 months post‐fire, and post‐fire reference data was measured ~12 months following fire. *n* = 5–42 plots.

**FIGURE 6:**
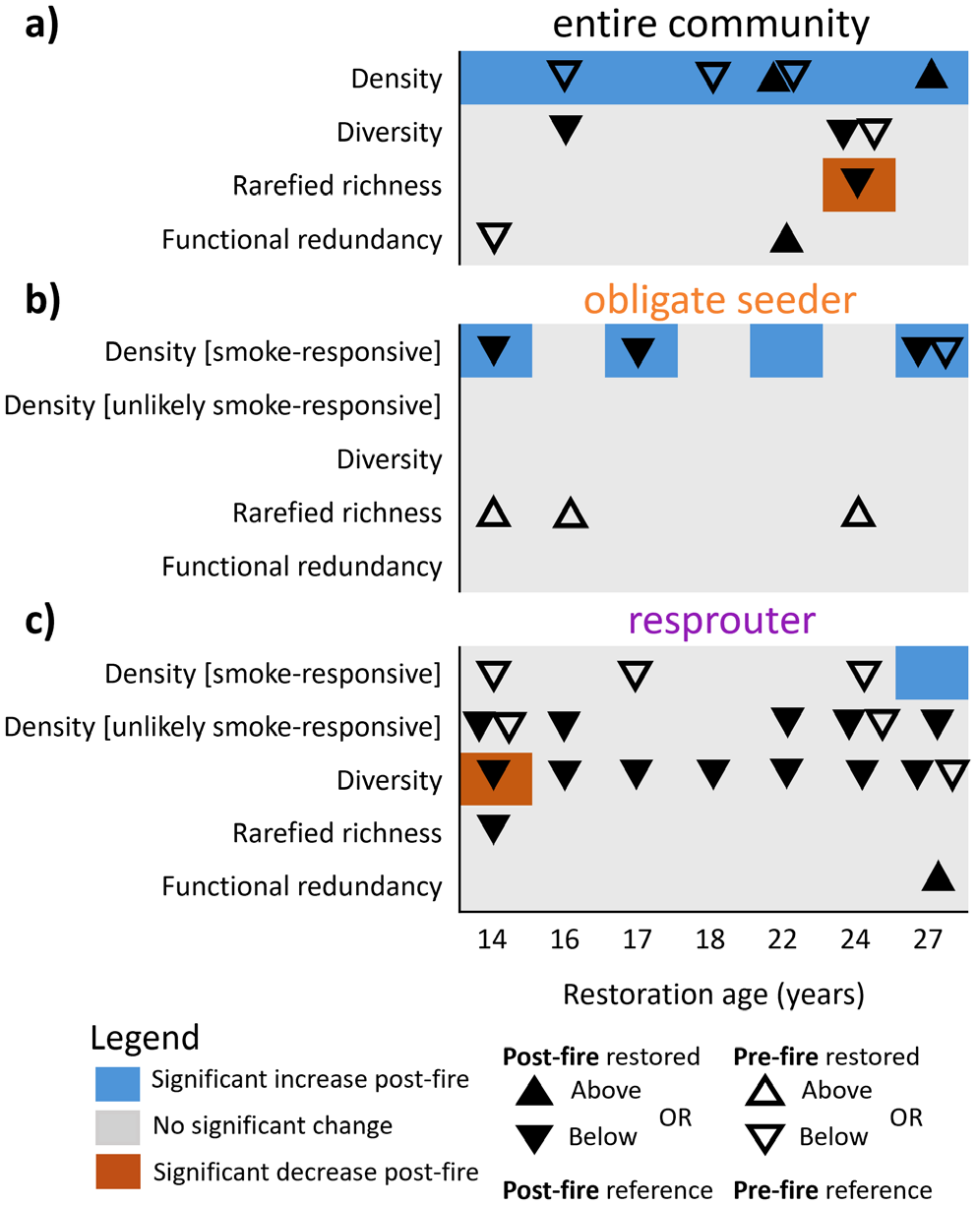
Summary for each resilience descriptor describing the change within restored sites (colours) and how restored sites compare to reference intact sites pre and post‐fire (symbols). Raw data is presented in Figure 5. Above or below refers to non‐overlap (in relevant direction) of restored and reference mean±95% CI in Figure 5.

For the entire community (annuals and native perennials), the average density of individuals per species pre‐fire was similar across all restoration ages and in a similar range to reference sites pre‐fire (Figure 5a). Following the fire, the entire community density significantly increased in all restoration ages, with restoration ages 22–27 having slightly higher density compared to restoration ages 14–18 years (5.8–9.7 vs. 12.2–17.5 individuals m^−2^; Figures 5a and 6).

Post‐fire diversity, rarefied richness, and functional redundancy of the entire community were similar to pre‐fire restored values across most of the restoration chronosequence (Figures 5d,g,j and 6). For these descriptors, in most cases, restored sites both pre‐ and post‐fire met reference values for the resilience descriptors, except for post‐fire diversity in ages 16 and 24, and post‐fire rarefied richness in age 24, which were all significantly lower than reference sites (Figure 6).

Obligate seeder density was similar pre and post‐fire in all restoration ages, except for smoke‐responsive densities in restoration ages 14, 17, 22, and 27, which were significantly higher post‐fire (Figures 5b and 6). For both resprouters and obligate seeders, the density of smoke‐responsive species was higher than that of unlikely smoke‐responsive species (2–7 vs. 0–4 seedlings m^2^ per species respectively; Figure 5b,c). For obligate seeders, despite significant increases in smoke‐responsive density following fire in four restoration ages, post‐fire density in three of these restoration ages was significantly less than that of reference Banksia woodland following fire (Figure 6). Resprouters had significantly lower unlikely smoke‐responsive density post‐fire in all ages compared to reference sites, except at 17 and 18 years (Figures 5c and 6).

Across the entire restoration chronosequence, obligate seeder diversity, rarefied richness and functional redundancy did not significantly change following fire and were typically similar to that of reference sites (Figures 5e,h,k and 6). For resprouting species, there was a significant decline in diversity in Age 14 following fire, with little change in diversity, rarefied richness, and functional redundancy after fire in restored sites (Figures 5f,i,l and 6). Post‐fire resprouter diversity in all restoration ages was significantly lower than that observed in reference Banksia woodland, with post‐fire rarefied richness in restoration Age 14 significantly less than that in reference Banksia woodland (Figures 5f,I and 6).

### 5.5 Pooled Resilience

**FIGURE 7:**
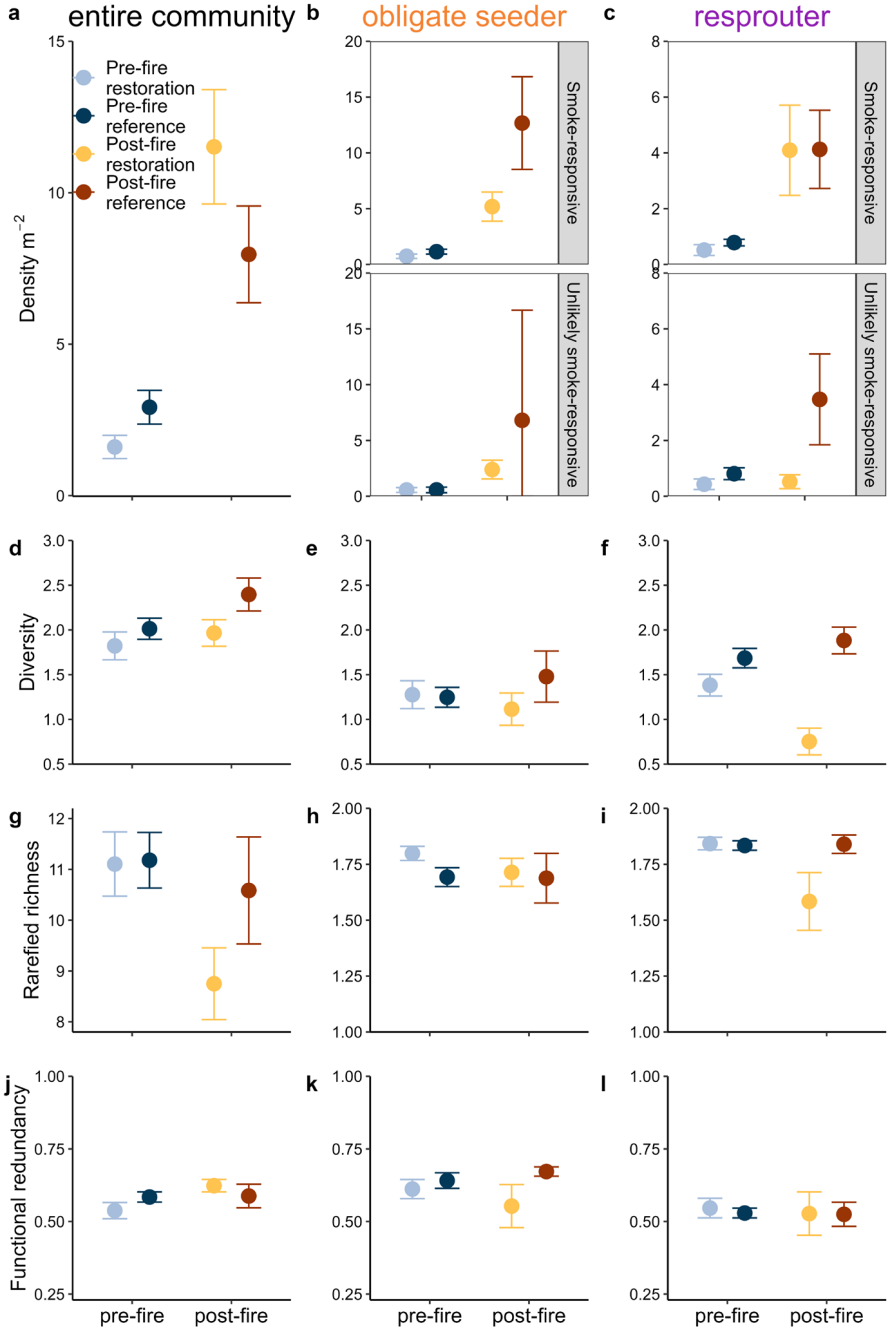
Means±95% CI values for pooled resilience for the entire community (annuals and native perennials) and perennial obligate seeder and resprouter density, diversity, rarefied richness and functional redundancy in Banksia woodland.

**FIGURE 8:**
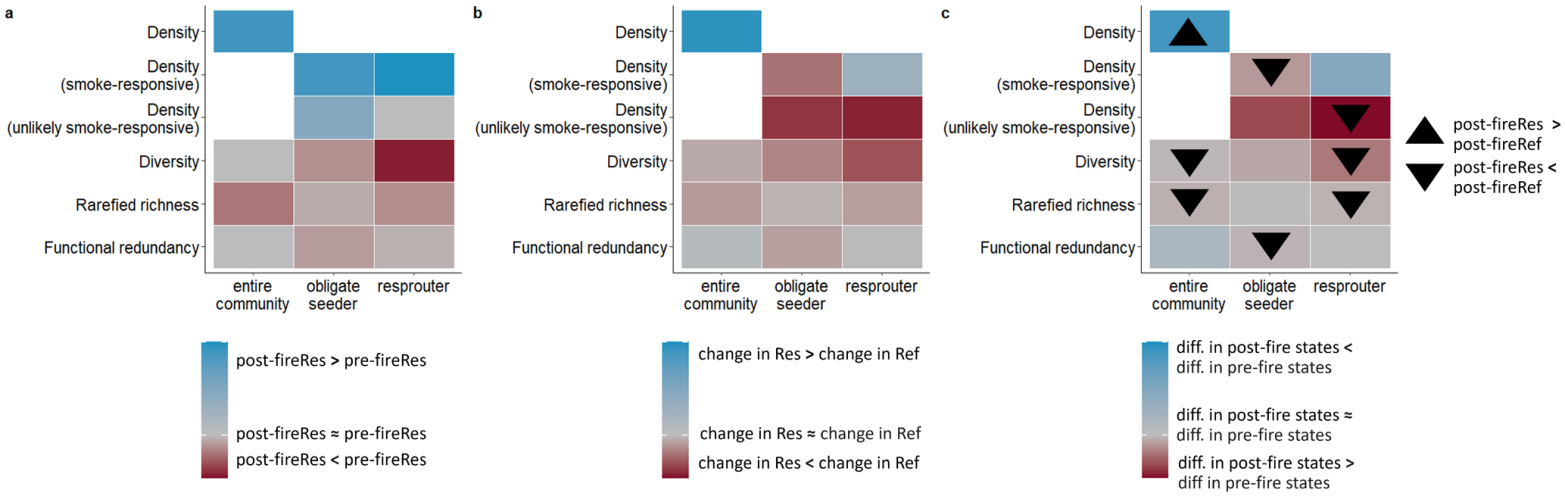
Amounts of change for pooled resilience (Figure 2) for the three pairwise assessments: (a) change in restored sites from fire, (b) change in restored sites regarding reference sites following fire, and (c) difference in post‐fire states (for restored and reference sites) compared to pre‐fire states (i.e., has fire shifted restored states closer to reference et states). Arrows in (c) show whether post‐fire restored states significantly differ from that in reference, based on Figure 7. Specific interpretations differ among the three comparisons (see legends), but broadly, blue tiles demonstrate a desirable response of restored sites, while red suggests an undesirable response of restored sites regarding resilience. Res = restored, Ref = reference, diff. = difference. Panels refer to A, B and C comparisons in Figure 2.

Following fire in restored sites, with restoration ages combined, density significantly increased for all assemblages except for unlikely smoke‐responsive resprouters, which did not change (Figures 7a–c and 8a). However, the change following fire in restored Banksia woodland was less than that in reference sites for unlikely smoke‐responsive density for both resprouters and obligate seeders (Figures 7b,c and 8b). Similarly, while smoke‐responsive obligate seeder density significantly increased following fire in restored sites, this amount of change was less than that observed in reference sites (Figures 7b and 8a,b).

Comparisons of change following fire in restored sites found that fire either had little effect or decreased diversity, rarefied richness and functional redundancy for all assemblages, with particularly high losses observed in the rarefied richness of the entire community and resprouter rarefied richness and diversity (Figures 7d–l and 8a). This is reflected when comparisons of change in restored sites are considered relative to those in reference sites, where the amount of change in restored sites was similar to, or less than that in reference sites (Figure 8b). High losses in resprouter diversity following fire in restored sites were also reflected by restored sites changing less than reference sites, where reference sites increased while restored sites significantly decreased (Figures 7f and 8b).

Following fire, the difference in density between restored and reference sites for the entire community and smoke‐responsive resprouters was less than that observed pre‐fire (i.e., fire shifted resprouter density closer to reference states; Figure 8c). The difference in unlikely smoke‐responsive density of both obligate seeders and resprouters following fire was more than that pre‐fire (i.e., fire shifted density further from reference states; Figure 8c). The amount and direction of change in pre‐fire and post‐fire restored and reference sites were similar for most measures of diversity, rarefied richness, and functional redundancy (Figure 8c). However, despite this, resilience descriptors were significantly lower in post‐fire restored states than those of reference states for 50% of descriptors for the entire community, 40% for obligate seeders, and 60% for resprouters (Figure 8c).

### 5.6 Consistency of Pooled Resilience Assessments

Across the three comparisons used to assess pooled resilience, comparisons utilising reference sites (Figure 8b,c) revealed differing levels of resilience compared to comparisons utilising change in restored sites only (Figure 8a), particularly for assessments of density. For example, obligate seeder smoke‐responsive density was found to increase post‐fire in restored sites (Figure 8a), but comparisons utilising reference data demonstrate that relative change in restored sites was substantially less than that of reference sites, while fire made restored and reference states more different than pre‐fire states (Figure 8b,c). For most assessments of diversity, rarefied richness and functional redundancy, resilience comparisons were typically similar, as demonstrated by similar colours (representing the amount and direction of change) among the three comparisons (Figure 8).

### 5.7 Resilience Based on Assemblage Composition

**FIGURE 9:**
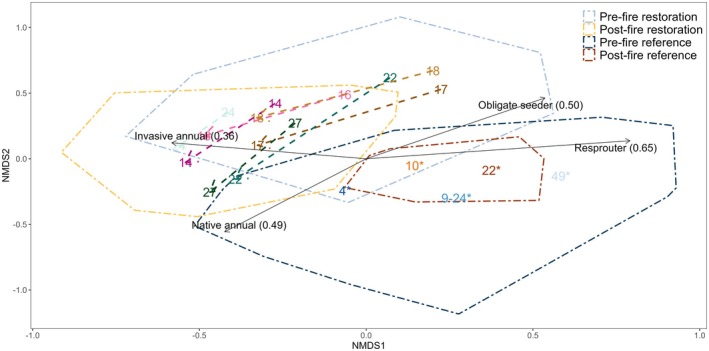
Axis 1 and 2 of a 3D NMDS of Hellinger transformed Bray–Curtis similarity of ecosystem types (polygons), age centroids, and a biplot of proportions of individuals per trait. Arrows link the pre‐fire assemblage to the post‐fire assemblage for each restoration age. Ages with * are reference Banksia woodland plots; numbers in brackets for species trait biplot are R^2^ values. Ecosystem type polygons surround all plots for that state; individual plots are not shown for ease of visualisation. Stress = 0.18. Axis three revealed more similarity between the reference and restored Banksia woodland compared to Figure 9.

**FIGURE 10:**
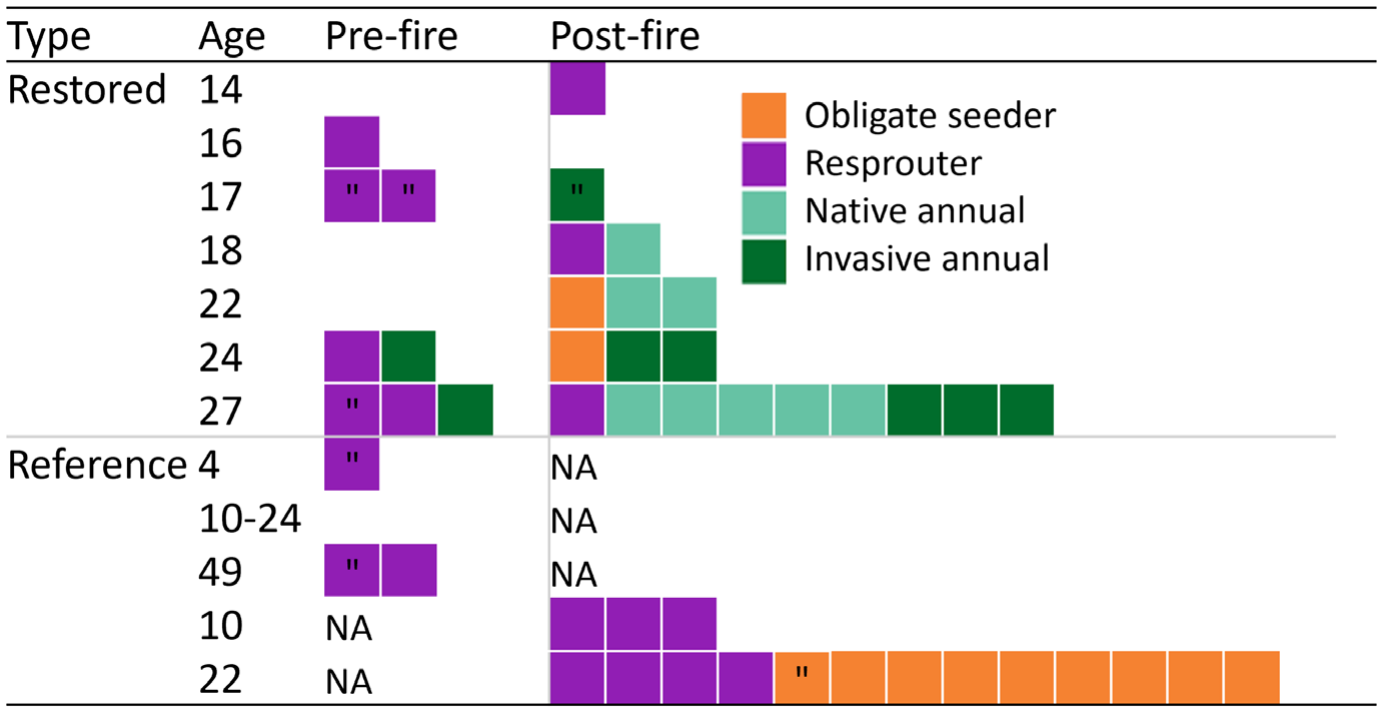
Counts by trait types of indicator species across the site ages. NA = group not assessed for reference sites as age not present, = species found in restored or reference sites only. See Appendix S2: Table S4 for species associated with each age and ecosystem type. Each coloured box refers to one species.

For some resilience descriptors, restored states were more similar to reference states after fire than before fire (Figure 8c). Yet community composition analyses revealed that post‐fire restored and reference sites were distinct in species composition as indicated by the lack of overlap in polygons (Figure 9). This was largely driven by differences in perennial species abundance (Figures 9 and 10). ISA revealed that post‐fire reference sites were entirely differentiated by perennial indicator species, while post‐fire restored sites were mostly differentiated by annual indicator species as 74% of indicator species were annuals (Figure 10; Appendix S2: Table S4). Similarly, NMDS revealed that perennial resprouters and obligate seeders were correlated with reference site composition, while annuals were correlated with restored sites (Figures 9 and 10). Overall, significant differences were observed among both restoration ages and between restored and reference site species composition (ANOSIM *R* = 0.29, *p* ≤ 0.001 & *R* = 0.34, *p* ≤ 0.001 respectively). The high density of the entire community in restoration age 27 is likely due to five native and three invasive annual species found as indicators in this age (Figure 10).

## 6 Discussion

Experimental fires across a 13‐year chronosequence of restored Banksia woodland resulted in fire impact being similar across restoration ages. Post‐fire regeneration at ~5 months and survival to ~17 months of seedlings and established resprouts were not influenced by restoration age, fire impact, or soil conditions, and mixed models were characterised by high amounts of unexplained variability.

Resilience of restored Banksia woodland to fire was suggested by some but not all the descriptors we assessed. For example, when comparing the change in restored sites pre‐ to post‐fire, the density of obligate seeders was similar or higher which suggests resilience in restored sites. However, these species groups demonstrated less resilience than reference sites as the amount and direction of change in restored sites did not always reflect that of reference Banksia woodland. Restored sites' resprouter diversity and rarefied richness significantly decreased post‐fire, which did not occur in reference sites. However, the diversity and functional redundancy of the entire plant community suggested community‐level resilience to fire was present given similar overall changes across restored and reference Banksia woodland sites. Comparison of changes using reference site data was key to assessing the resilience of restored sites to fire, with pre‐ and post‐fire restored sites distinct from reference sites due to differing species composition and trait dominance. Specifically, annuals were dominant in restored sites while perennials were dominant in reference Banksia woodlands.

### 6.1 Post‐Fire Recruitment and Resprouting Survival Was Not Influenced by Restoration Age or Legacies

Following fire, resprouting and seedling recruitment occurred across all restoration ages in restored Banksia woodland. We found that survival rates of perennial species seedlings ranged from 15% to 20% after one post‐fire summer where seedling mortality is typically at its highest. This is similar to survival rates after the first summer following direct seeding of six species to investigate plant facilitation at the same site (survival across all species = 18.2%–21.6%; species level survival = 0.8%–54.3%; Svejcar 2020). Slightly higher survival values after the first summer have also been found in Banksia woodland following restoration (~20%–55%; Standish et al. 2012) and following fire in post‐mining restored sclerophyllous species‐rich Kwongan shrublands ~250 km north of Hanson (survival = ~24%), which shares a similar suite of species (Herath et al. 2009).

Resprouting species were the most speciose trait group in restored sites, and we found that approximately 43% of resprouter individuals present before fire had resprouted and survived to ~17 months post‐fire. We found lower established resprouter survival than post‐fire studies in post‐mining restored and intact Kwongan (mean = 52 vs. 96% and 85% respectively for these studies; Herath and Lamont 2009; Enright et al. 2011). Similarly, Cowan, Fontaine, et al. (2023) found restored sites resprouter survival to range from 3% to 92%, further highlighting the variation in resprouting success among species and resprouter types.

Across the restoration chronosequence, we found that overall germination of perennial species was not significantly influenced by restoration age. This result is consistent with findings in Cowan, Miller, et al. (2023) and from intact Mediterranean climate woodlands in southeastern Australia (Chick et al. 2015). We found no evidence of increased resprouting success in older restoration ages, consistent with research in mine site restored and intact Kwongan where resprouter survival varied with fire intervals (Herath and Lamont 2009; Enright et al. 2011). Species‐specific responses and other factors including pre‐fire plant size may be more important for resprouting success than restoration age (Cowan, Fontaine, et al. 2023).

We found no effect of fire impact, soil compaction and field capacity on post‐fire regeneration and survival despite their impact on resprouters' post‐fire responses observed in Cowan, Fontaine, et al. (2023) and other restoration projects (e.g., Herath et al. 2009; Herath and Lamont 2009; Riviera et al. 2021). While soil variables have been found to influence plant establishment following restoration (e.g., Holmes 2001; Rokich et al. 2001; Timsina et al. 2022), they seem to have inconsistent effects on responses to fire. We found no effect at the plant assemblage scale even though effects at the plant population scale were identified in Cowan, Fontaine, et al. (2023). Therefore, we suggest further investigation of the effects of soil attributes on plant responses to fire, potentially through measurement at finer scales.

### 6.2 Resprouters Demonstrated Variable Early‐Stage Resilience to Fire

Plant age affects the development of resprouter organs and resprouting success following fire (Herath and Lamont 2009; Enright et al. 2011; Cowan, Fontaine, et al. 2023). When comparing pre‐ vs. post‐fire states, we found reduced diversity in restoration ages 14 and 16 years, and reduced rarefied richness in restoration age 14 compared to reference sites. This suggests that 14–16 years could be too short of a time for plants to develop resprouting organs in restored sites (noting we were unable to assess ages younger than 14 years). These restoration ages are substantially younger than plants in intact sites which may be many decades to centuries old (Merwin et al. 2012; Enright et al. 2014; Miller et al. 2024). This interpretation is consistent with a study of resprouter recovery to fire in post‐mining sites of a similar age in the same region (Herath and Lamont 2009).

In our assessments of pooled resilience (restoration ages combined) we found that the density of unlikely smoke‐responsive resprouters and resprouter diversity change following fire in restored sites did not reflect that of reference sites suggesting that the resilience between restored and reference Banksia woodlands differs. Additionally, for these species groups fire appeared to shift restored states further from reference states. However, restored sites' rarefied richness decreased following fire, but the change was somewhat similar to reference sites, and fire did not appear to shift restored and reference states further from each other. This suggests that for rarefied richness, resilience between restored and reference sites was similar.

To better understand resprouter resilience to fire, additional research should further explore drivers of resprouting success by considering factors including carbohydrate storage and bud‐bank availability (Clarke et al. 2013; Ott et al. 2019) using a trait‐based or species‐specific approach (Cowan et al. 2021). Additionally, this study revealed that the perennial species (particularly resprouters) found in restored Banksia woodlands were only a subset of those found in the reference Banksia woodland. For restoration goals based on achieving similar species and trait composition to reference sites, further knowledge is needed to promote the successful establishment and survival of resprouters following topsoil transfer.

Resprouter densities, rarefied richness, and diversity in restoration ages 14 and 16 reduced following fire or were lower than reference sites following fire, suggesting resilience is incomplete compared with that of reference sites which did not significantly reduce following fire. Subsequent management may be required to facilitate the return of resprouter species. For example, planting and seeding may be required to promote similar species composition between restored and reference sites if this reflects restoration goals. This should be completed during or a few years after topsoil transfer when baseline surveys have been able to determine if composition differs. Resprouting species are dominant in Banksia woodland, and their persistence post‐fire is a key metric of their resilience as it demonstrates their stability. Therefore, it may be appropriate to exclude fire from restoration sites until reference levels of resprouters have been attained, or plants have been able to develop sufficient resprouting capacity to avoid undesirable trajectories. However, comparisons of trajectories with and without fire are required to understand changes through time and the potential role of fire in changing ecosystem states.

### 6.3 Variables Describing Obligate Seeders Suggested Resilience to Fire

We found that restoration age typically did not impact the recovery of obligate seeders to fire, across the variables we measured, likely reflecting high seedbank density and richness in restoration ages > 13 years (Cowan, Miller, et al. 2023). We found that obligate seeder density post‐fire was less than that in reference Banksia woodland, but this did not result in substantial differences in diversity, rarefied richness, or functional redundancy of this trait group in restored sites following fire. Change following fire was similar in restored and reference sites for rarefied richness and slightly dissimilar for diversity and functional redundancy, and fire did not shift obligate seeder states further from reference states, thereby suggesting resilience. We suggest that higher seed quantities and quicker growth rates in obligate seeders compared to resprouters (Pate et al. 1990; Lamont and Wiens 2003; Cowan, Miller, et al. 2023) enable these species to rapidly accumulate a seedbank that can respond to fire earlier in the restoration trajectory.

### 6.4 Important Considerations for the Assessment of Resilience in Restored Ecosystems

Despite the importance of resilience for restoration success, its quantification remains difficult (Standish et al. 2014; Cowan et al. 2021), so we assessed the resilience of restored Banksia woodland plant assemblages using three approaches, with four resilience descriptors and relating to two levels of biological organisation (community vs. population). These were assessed at both the species level and with species grouped by traits. Broadly, we found most resilience descriptors (i.e., change in diversity, rarefied richness, or functional redundancy) often suggested resilience as there was little change pre‐ post fire in restored sites, and restored states were often comparable to reference states, except for resprouters. Analysis of community composition using ordination and ISA however, revealed more distinct changes between restored and reference sites that were not apparent in the resilience descriptors. Specifically, restored sites did not contain a large component of resprouters observed in reference sites. Due to the difficulty in successfully establishing all plant species in restoration projects (e.g., Riviera et al. 2021; Shackelford et al. 2021; De Vitis et al. 2022; Gerrits et al. 2023), it is important to incorporate assessments of community composition comparing restored and reference states. Community‐level analyses can help to identify specific species and trait groups that may respond poorly to a disturbance and streamline future research and management (Cowan et al. 2021), so a multi‐faceted approach to assessing resilience is required.

The inclusion of reference Banksia woodland data was also essential for understanding if resilience was similar in restored and reference sites. Specifically, assessments of pooled resilience revealed that while values changed following fire in restored sites, the amount of, and direction of change did not reflect that of reference sites in some cases such as density. Fire caused a substantial change in restored sites, but the inclusion of reference data reveals that fire did not shift restored sites further from reference sites, as the difference in post‐fire and pre‐fire states was similar, except for unlikely smoke‐responsive species. This is apparent in the rarefied richness of both the entire community and resprouter group, and functional redundancy among obligate seeders. Where possible, we suggest the inclusion of both pre‐and post‐disturbance reference data to allow for assessments of resilience in restored sites, as similarity in states (e.g., density, diversity) and change following fire are key elements of resilience in restoration projects. We also acknowledge that for some reference site groupings, there was low replication, and that the inclusion of a wider range of reference sites may have demonstrated more variability in reference Banksia woodland and influenced interpretations of the resilience of restored sites. Additionally, this work does not quantify the resilience of other processes to fire such as water infiltration, nutrient cycling, and plant–soil feedbacks, which could drive community responses (Miller et al. 2017).

Resilience proxies, including those that are based on species functions, are proving powerful at assessing resilience as they allow for further assessment of changes in ecosystem function and future resilience (Biggs et al. 2020; Standish et al. 2021; Cowan et al. 2021). Through our assessment of functional redundancy, we typically found little change following fire in restored sites and reference sites. This was true for resprouters also: despite their declines in diversity and rarefied richness post‐fire, and differences in density, diversity and rarefied richness compared to reference states, ecosystem functioning was maintained (i.e., species lost following fire were not performing specialised functions). This aligns with research by Carrick and Forsythe (2020) and Standish et al. (2021) that found species composition alone accounts for very little of the variation of ecosystem function, which may be more apparent in highly diverse sites where species share functional roles (Holmes and Richardson 1999; Gallagher et al. 2013; Araújo and Conceição 2021). While our findings suggest resilience based on the recovery of functional redundancy following fire, it is important to note that such indices are heavily dependent on the trait data used, which can be difficult to obtain and may not account for high levels of trait variability (e.g., Moreira et al. 2012; Mitchell et al. 2020; Westerband et al. 2021). Therefore, we advise caution regarding the development of restoration goals and management plans based solely on functional proxies due to the mixed importance of species' roles.

It is important to note the potential effects of the unfavourable weather conditions observed during the first summer after fire in this study (when plants were ~8–11 months old). During this period, maximum summer temperatures were ~0.9°C higher than the average, and a record number of days over 40°C occurred (Logan 2022), which may have increased the mortality of seedlings and established resprouts (Pratt et al. 2014; Stewart et al. 2021; Nolan et al. 2021; Bendall et al. 2022; Salesa et al. 2022) but see Enright et al. (2014) and Parra and Moreno (2018). Increased mortality in the established but unburnt restoration was observed at Hanson following this extreme summer also (E. Cowan, pers. obs., March 2022), highlighting the potential vulnerability of these restored sites to drier and warmer conditions. Uncertainty of the effects of extreme post‐fire weather conditions on plant responses makes it difficult to determine to what extent our results were influenced, so post‐fire responses following “average” summer conditions should not be expected to be different. Furthermore, resilience to wildfire may differ from that of experimental fires, with higher fire intensity and younger ages potentially being burnt during wildfire than what occurred in this study. Additionally, assessments of younger or older restoration ages than was surveyed may have yielded different interpretations of resilience, particularly in reference to minimum or maximum tolerable fire intervals of restored sites.

In restored Banksia woodland a range of descriptors were assessed to understand resilience to fire. For obligate seeder and resprouter species groups, density and diversity measures in restored sites did not reflect those of reference sites suggesting differences in restored and reference site resilience. Additionally, resprouters demonstrated losses of diversity and rarefied richness after fire in restoration Age 14, and more broadly had lower levels of resilience in most descriptors than reference Banksia woodland. Community composition differed substantially as annual species drove composition in restored sites, and perennials drove the reference Banksia woodland. Resprouters in restored Banksia woodland were a subset of those in reference Banksia woodland due to initial restoration establishment rather than fire responses, and their underrepresentation reduces their resilience to fire. Therefore, research and management focus on promoting the successful establishment of resprouters will increase compositional similarity. The inclusion of reference data is necessary to interpret whether changes in restored sites are desirable or not. Assessing resilience using a variety of variables is encouraged to fully describe the disturbance response of restoration projects.

## Author Contributions

**Ebony L. Cowan:** conceptualization (equal), data curation (lead), formal analysis (lead), investigation (lead), methodology (equal), project administration (lead), writing – original draft (lead), writing – review and editing (lead). **Rachel J. Standish:** conceptualization (equal), data curation (supporting), investigation (equal), methodology (equal), supervision (equal), writing – original draft (supporting), writing – review and editing (supporting). **Ben P. Miller:** conceptualization (equal), data curation (supporting), investigation (equal), methodology (equal), supervision (equal), writing – original draft (supporting), writing – review and editing (supporting). **Russell G. Miller:** data curation (supporting), formal analysis (supporting), investigation (supporting), writing – original draft (supporting), writing – review and editing (supporting). **Willa P. Veber:** conceptualization (supporting), data curation (equal), methodology (supporting), writing – original draft (supporting), writing – review and editing (supporting). **Joseph B. Fontaine:** conceptualization (equal), data curation (supporting), formal analysis (supporting), investigation (equal), methodology (equal), supervision (equal), writing – original draft (supporting), writing – review and editing (supporting).

## Conflicts of Interest

The authors declare no conflicts of interest.

## Supporting information


**Appendix S1:** ece372445‐sup‐0001‐AppendixS1.docx.

## Data Availability

The data and analysis code that supports the findings of this study are available in Mendeley data repository at data.mendeley.com/datasets/b922g299bh/1. The code used is not novel.
