# Supplementary material for: Ecological Resilience of Restored Mediterranean‐Climate Woodlands to Experimental Fire
Source: Ecol Evol. 2025 Nov 16;15(11):e72445. doi: 10.1002/ece3.72445 (PMC12620566; doi:10.1002/ece3.72445)
Supplement: Supplementary file 1 — Appendix S1: ece372445‐sup‐0001‐AppendixS1.docx. [file ECE3-15-e72445-s001.docx]

**Journal name:**Ecology and Evolution

**Title**:

Ecological resilience of restored Mediterranean-climate woodlands to experimental fire

**Authors:**

Ebony L. Cowan^1,2^, Rachel J. Standish^1^, Ben P. Miller^2^, Russell G. Miller^1,2^, Willa P. Veber^1^, Joseph B. Fontaine^1^

**Authors affiliations**

^1^ School of Environmental and Conservation Sciences, Murdoch University, Murdoch 6150, Western Australia, Australia

^2^ Kings Park Science, Biodiversity and Conservation Science, Department of Biodiversity, Conservation and Attractions, 1 Kattidj Close, Kings Park 6005, Western Australia, Australia

**Corresponding author:** Rachel Standish, School of Environmental and Conservation Sciences, Murdoch University, Murdoch 6150, Western Australia, Australia

**Email:** r.standish@murdoch.edu.au

**Open Research Statement:** The data and analysis code that supports the findings of this study are available in Mendeley data repository at data.mendeley.com/datasets/b922g299bh/1. The code used is not novel.

**Appendix S1: Methods**


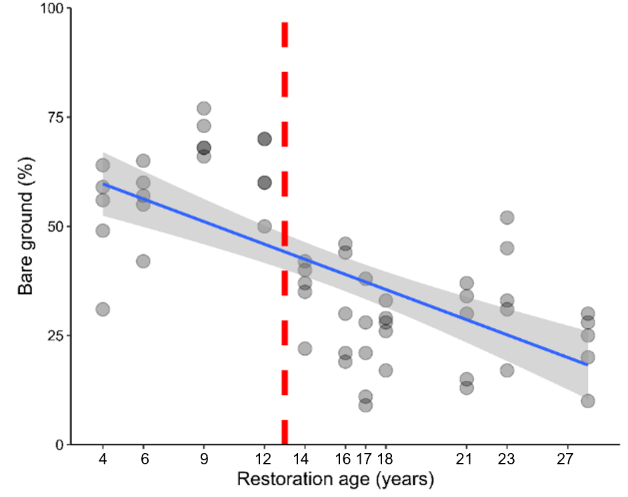


**Figure S1:** Pre-fire bare ground cover (%) against restoration age. Ages younger than the red line did not have fire attempted.

**Table S1:** Reference Banksia woodlands site information and distances from Hanson. Post-fire data was measured one year following fire, with time since previous fire recorded in the table. Age groups reflect groups pooled for analysis of resilience within ages.

| Measurement type | Age (years) | Number of sites | Number of plots | Distance from Hanson (km) |
| --- | --- | --- | --- | --- |
| Pre-fire | 4 | Two | 15 | 1.3–4.5 |
|  | 9-24 | Six | 42 | 3.2–8.5 |
|  | 49 | One | 7 | 8.5 |
| Post-fire | 10 | One | 5 | 4.5 |
|  | 22 | One | 7 | 6 |

**Supplementary text S1: Data structure for responses to fire analyses**

For regeneration responses (~5 months post-fire), we determined post-fire annual and perennial seedling densities by species, grouped by plot and restoration age. Annuals were separated by their origin (native vs invasive) as invasive species can influence restoration success, while native perennial seedlings were separated according to the smoke-responsive status of their seedbank (likely vs unlikely smoke-responsive; 73 and 27% of perennial species in restored sites respectively; Figure 2). This allowed us to quantify the unlikely smoke-responsive component of Banksia woodlands (dominated by heat-responsive and species with no seed dormancy) separately which was unable to be quantified by Cowan et al., (2023b). Within perennial seedlings’ smoke-responsive status, we further separated species based on their fire-response type (obligate seeder vs resprouter) as the development of these attributes may differ due to plant longevity and resource allocation (Enright et al., 2014; Pausas & Keeley 2014).

The resprouting response at ~5 months post-fire was calculated using the proportion of post-fire established resprouts (those that had resprouted by ~5 months post-fire) compared to pre-fire established plants that were capable of resprouting (resprouter fire-response type) and was grouped by species and clonality type. Separating species into clonal (49% of resprouting species) and non-clonal (51%) species allowed us to assess if drivers of resprouting differ as suggested by Cowan et al., (2023a). Common resprouter organ types for clonal species included rhizomes and root suckers, while non-clonal species typically had epicormic buds or lignotubers (Clarke et al., 2013).

**Table S2:** Databases used for plant trait data.

| Trait | Source |
| --- | --- |
| Fire response type (resprouter vs obligate seeder), growth category & seed storage mode | Veber et al., (2019; unpublished data) |
| Origin & longevity | Western Australian Herbarium (1998–) |
| Smoke-responsive status | Sweedman & Merritt (2006); Offord & Meagher (2009); Baskin & Baskin (2014); Stevens et al., (2016) |
| Clonal type (as per Clarke et al., 2013) | Wilson et al., (2010); Pausas et al., (2018); Veber et al., (2019; unpublished data) |
| Seed mass | Falster et al., (2021); Lewandrowski & Turner, unpublished data |
| Specific leaf area | Falster et al., (2021) |
| Microbial associations | Tsakalos et al., (2020) |


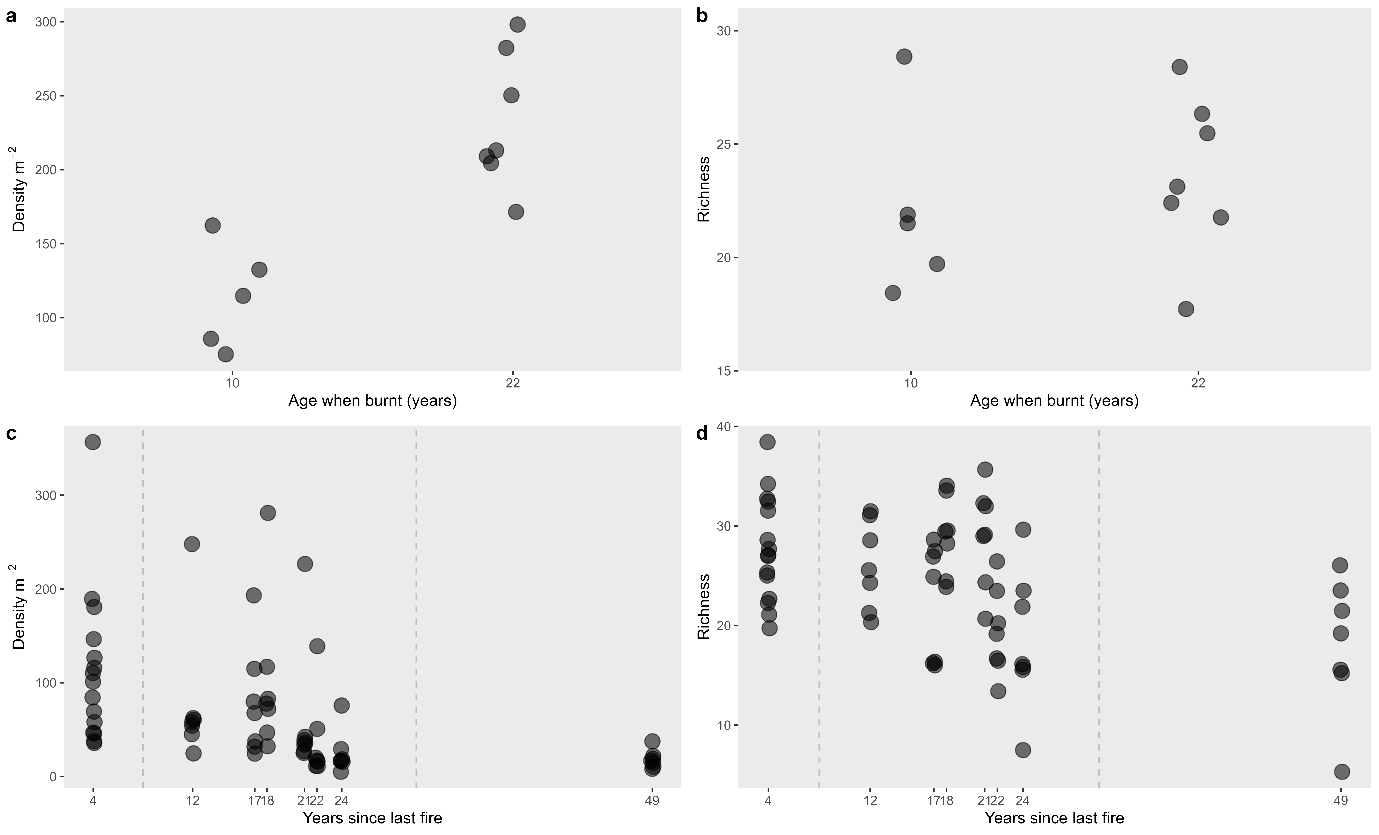


**Figure S2:** Post-fire (a.b) and pre-fire (c,d) reference sites densities and richness against years since last disturbance. The dotted lines on c & d indicate where different aged sites have been split for analysis.

**Supplementary text 2: Modelling drivers of post-fire responses in restored Banksia woodlands**

Using generalized linear mixed models (GLMMs), we sought to determine the effects of various drivers on post-fire regeneration (~5 months post-fire) and survival (~17 months post-fire) of perennial seedlings, annuals (~5 months only) and established resprouts (Figure 2). Regardless of smoke-responsive status, perennial seedlings were modelled together, and both clonal and non-clonal established resprouts were also modelled together as there was little difference between the trait types in data exploration and this helped to increase sample sizes.

For all models, restoration age was included as a continuous predictor. The following traits for responses to fire were modelled as fixed effects: fire response type (obligate seeder or resprouter) and smoke-responsive seedbank status (likely smoke responsive or unlikely) for perennial seedlings, origin for annuals (native vs invasive), and clonality for established resprouts (clonal or not; Figure 2). For models of post-fire regeneration (~5 months post-fire), perennial and annual seedling models also included the average percent fire coverage of each subplot burnt per plot as a continuous predictor as this was where the seedling response was assessed. For models of established resprouts, fire coverage of each subplot was substituted for fire severity per plot (continuous predictor) as this describes how many plants were burnt which is necessary for resprouting. Fire impact variables were not included in models of ~17 month (survival) responses as we expected soil conditions to be more important for survival than fire impact. Therefore, field capacity per plot was included as a continuous predictor in models for both perennial seedlings and established resprouts proportions of survival. Additionally, average soil compaction per plot at 10 cm was included as a continuous predictor in models for seedlings, and 30 cm compaction (continuous predictor) for resprouts, with the depth of compaction matching expected rooting depths. Interaction terms were added between restoration age and fire response type (perennial seedlings), clonality (established resprouts), and origin (annuals) to assess whether responses along the restoration age chronosequence varied for species in different classes of these variables. Interaction terms were also included between fire impact and plant traits described above to assess if the responses among trait groups varied with the amount of fire.

Models of perennial and annual seedling counts at ~5 months were constructed using a negative binomial distribution with a log-link due to overdispersion observed in the data (McCullagh & Nelder 1989). All proportion data (i.e., established resprouts at ~5 & 17 months, and perennial seedlings survival at ~17 months) were modelled using a beta distribution (Ospina & Ferrari 2010; Geissinger et al., 2022). All models included separate random effects of species and restoration age to account for potential unexplained differences among groups. Continuous predictors were standardized by subtracting the mean of each value and dividing by the standard deviation to reduce estimate biases (Zuur et al., 2009; Schielzeth 2010).

All models of post-fire responses were conducted in the R package *glmmTMB* (Brooks et al., 2017), with the *performance* (Lüdecke et al., 2021), *MuMIN* (Barton 2023) and *rcompanion* (Mangiafico 2023) packages used to assess collinearity of covariates, normality and heterogeneity of model residuals, and model performance. The presented models include all predictors as removing predictors from initial models did not result in a change to Akaike Information Criteria (AIC; Akaike 1973) scores (i.e., all models within 2 AIC; Burnham & Anderson 2004). To prevent overfitting, we removed predictors that had a variance inflation factor (VIF) of >5 (Alauddin & Nghiemb 2010). For count models using a negative binomial distribution, trigamma R^2^ was reported due to its highly accurate estimates of the variance in models using a log-link function (Nakagawa et al., 2017), while Cox and Snell’s (1989) pseudo-R^2^ was estimated for proportional models using a beta distribution as per Geissinger et al., (2022). In some cases, random effect estimates were approximately 0 resulting in no difference between the conditional and marginal R^2^ (Schielzeth et al., 2020). *P values* were adjusted using the ‘Holm-Bonferroni’ adjustment to account for multiple statistical tests (Holm 1973) using the ‘p.adjust’ function in the *stats* package (R Core Team 2020). The adjusted *p values* are reported.

**Table S3:** Traits used in functional redundancy analysis and their reasoning. For categorical traits, the potential factors and number of species (pooled across restored and reference plant communities) are included. Traits with * were not included for calculation of obligate seeders and resprouters functional redundancy. Invasive perennials are not included as they were removed from analysis.

| Trait (number of species missing trait value) | Reasoning | Factors | Number of species |
| --- | --- | --- | --- |
| Fire response* | Main post-fire response mechanism | Resprouter | 89 |
|  |  | Obligate seeders (includes annuals) | 88 |
| Longevity* | Relates to growth and regeneration | Perennial | 130 |
|  |  | Annual | 47 |
| Species origin* | Relates to growth, dispersal and restoration success | Native | 158 |
|  |  | Invasive | 19 |
| Growth form | Ability to compete for light, ecosystem structure | Herb | 64 |
|  |  | Shrub | 56 |
|  |  | Tussock | 24 |
|  |  | Grass | 16 |
|  |  | Tree | 6 |
|  |  | Succulent | 6 |
|  |  | Geophyte | 3 |
|  |  | Palmoid | 2 |
| Smoke-responsive status | Relates to dormancy responses | Likely | 138 |
|  |  | Unlikely | 39 |
| Seed storage location | Relates to regeneration | Soil | 160 |
|  |  | Canopy | 15 |
|  |  | None | 2 |
| Microbial associations (5) | Nutrient acquisition and soil microbial composition | Arbuscular-Ectomycorrhizal | 115 |
|  |  | Root microbial | 43 |
|  |  | Ericoid | 14 |
| Seed mass (69) | Impact dispersal, persistence, establishment, survival | Numerical |  |
| Specific leaf area (110) | Relates to photosynthesis, growth, water balance | Numerical |  |


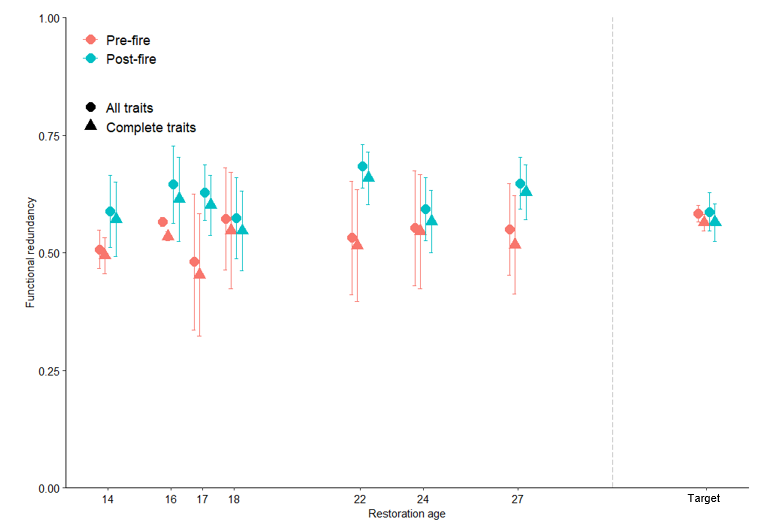


**Figure S3:** Mean and 95%CI of functional redundancy for each restoration age and measurement type (colours) for all traits and complete traits used in calculation. Reference data is to the left of the dashed grey arrow. Complete traits are those with >95% of species with a trait value (i.e., excludes SLA and seed mass).

**Appendix S2: Results**

**Table S1:** List of species found in each data type, and their trait types. For resprouters C=clonal, NC=non-clonal. * in life history type = unlikely smoke responsive.

|  |  |  | Restoration | | Reference | |  |
| --- | --- | --- | --- | --- | --- | --- | --- |
| Species |  |  | Pre-fire | Post-fire | Pre-fire | Post-fire | Life history type |
| acachueg | *Acacia huegelii* | Fabaceae | ✓ |  | ✓ |  | Obligate seeder * |
| acacpulc | *Acacia pulchella* | Fabaceae | ✓ | ✓ | ✓ |  | Obligate seeder * |
| adencygn | *Adenanthos cygnorum* | Proteaceae | ✓ | ✓ | ✓ | ✓ | Obligate seeder |
| airacary | *Aira caryophyllea* | Poaceae | ✓ |  | ✓ | ✓ | Invasive annual |
| airacupa | *Aira cupaniana* | Poaceae |  |  | ✓ |  | Invasive annual |
| alexnite | *Alexgeorgia nitens* | Restionaceae | ✓ | ✓ | ✓ | ✓ | Resprouter (C) |
| allohumi | *Allocasuarina humilis* | Casuarinaceae | ✓ | ✓ | ✓ |  | Resprouter (NC) * |
| amphturb | *Amphipogon turbinatus* | Poaceae |  |  | ✓ | ✓ | Resprouter (C) |
| andehete | *Andersonia heterophylla* | Ericaceae |  |  | ✓ | ✓ | Obligate seeder |
| anighumi | *Anigozanthos humilis* | Haemodoraceae | ✓ | ✓ | ✓ |  | Resprouter (C) |
| anigmang | *Anigozanthos manglesii* | Haemodoraceae | ✓ | ✓ | ✓ |  | Obligate seeder |
| arnoprei | *Arnocrinum preissii* | Hemerocallidaceae | ✓ |  | ✓ | ✓ | Resprouter (C) |
| astrpall | *Astroloma pallidum* | Ericaceae |  |  | ✓ |  | Obligate seeder |
| astrxero | *Astroloma xerophyllum* | Ericaceae | ✓ | ✓ | ✓ | ✓ | Obligate seeder |
| austcomp | *Austrostipa compressa* | Poaceae | ✓ | ✓ | ✓ | ✓ | Native annual |
| austflav | *Austrostipa flavescens* | Poaceae | ✓ | ✓ |  |  | Resprouter (NC) |
| avenbarb | *Avena barbata* | Poaceae | ✓ | ✓ |  |  | Invasive annual |
| bankatte | *Banksia attenuata* | Proteaceae | ✓ | ✓ | ✓ | ✓ | Resprouter (NC) * |
| bankilic | *Banksia ilicifolia* | Proteaceae | ✓ |  | ✓ |  | Resprouter (NC) * |
| bankmenz | *Banksia menziesii* | Proteaceae | ✓ | ✓ | ✓ | ✓ | Resprouter (NC) * |
| beaueleg | *Beaufortia elegans* | Myrtaceae | ✓ | ✓ | ✓ | ✓ | Obligate seeder |
| boropurd | *Boronia purdieana* | Rutaceae |  |  | ✓ |  | Obligate seeder |
| bororamo | *Boronia ramosa* | Rutaceae | ✓ | ✓ | ✓ |  | Obligate seeder |
| bosserio | *Bossiaea eriocarpa* | Fabaceae | ✓ | ✓ | ✓ | ✓ | Obligate seeder * |
| brizmaxi | *Briza maxima* | Poaceae | ✓ | ✓ | ✓ | ✓ | Invasive annual * |
| burccong | *Burchardia congesta* | Colchicaceae | ✓ |  | ✓ | ✓ | Resprouter (C) * |
| calabrev | *Calandrinia brevipedata* | Portulacaceae |  |  | ✓ |  | Native annual |
| calacorr | *Calandrinia corrigioloides* | Portulacaceae |  | ✓ | ✓ |  | Native annual |
| calaflav | *Caladenia flava* | Orchidaceae |  |  | ✓ | ✓ | Resprouter (C) * |
| calagran | *Caladrinia granulifera* | Portulacaceae |  | ✓ | ✓ |  | Native annual |
| calalini | *Calandrinia liniflora* | Montiaceae |  |  | ✓ |  | Native annual |
| calyangu | *Calytrix angulata* | Myrtaceae | ✓ |  | ✓ |  | Resprouter (NC) |
| calyflav | *Calytrix flavescens* | Myrtaceae |  |  | ✓ | ✓ | Obligate seeder |
| calyfras | *Calytrix fraseri* | Myrtaceae | ✓ |  | ✓ | ✓ | Obligate seeder |
| calysapp | *Calytrix sapphirina* | Myrtaceae |  |  |  | ✓ | Obligate seeder |
| calystri | *Calytrix strigosa* | Myrtaceae |  |  | ✓ |  | Resprouter (NC) |
| cassytha | *Cassytha sp.* | Lauraceae | ✓ |  | ✓ | ✓ | Obligate seeder * |
| carpedul | *Carpobrotus edulis* | Aizoaceae | ✓ | ✓ | ✓ | ✓ | Invasive perennial |
| centdrum | *Centrolepis drummondiana* | Centrolepidaceae |  |  | ✓ |  | Native annual |
| centglab | *Centrolepis glabra* | Centrolepidaceae |  | ✓ | ✓ | ✓ | Native annual |
| conoacul | *Conostylis aculeata* | Haemodoraceae | ✓ | ✓ | ✓ | ✓ | Resprouter (C) |
| conoaure | *Conostylis aurea* | Haemodoraceae |  |  | ✓ |  | Resprouter (C) |
| conocant | *Conostylis canteriata* | Haemodoraceae |  |  | ✓ |  | Resprouter (C) |
| conojunc | *Conostylis juncea* | Haemodoraceae | ✓ |  | ✓ | ✓ | Resprouter (C) |
| conopend | *Conostephium pendulum* | Ericaceae |  |  | ✓ |  | Resprouter (NC) |
| conoprei | *Conostephium preisii* | Ericaceae |  |  | ✓ |  | Resprouter (NC) |
| conoseti | *Conostylis setigera* | Haemodoraceae |  |  | ✓ |  | Obligate seeder |
| conostoe | *Conospermum stoechadis* | Proteaceae | ✓ | ✓ | ✓ |  | Resprouter (NC) |
| conybona | *Conyza bonariensis* | Asteraceae |  | ✓ |  |  | Invasive annual |
| corymicr | *Corynotheca micrantha* | Antheriaceae |  |  | ✓ |  | Resprouter (C) |
| crascolo | *Crassula colorata* | Crassulaceae | ✓ | ✓ | ✓ | ✓ | Native annual |
| crasexse | *Crassula exserta* | Crassulaceae |  |  | ✓ | ✓ | Native annual |
| cronking | *Croninia kingiana* | Ericaceae | ✓ |  | ✓ |  | Obligate seeder |
| dampline | *Dampiera linearis* | Goodeniaceae | ✓ | ✓ | ✓ | ✓ | Resprouter (C) |
| dasybrom | *Dasypogon bromeliifolius* | Dasypogonaceae | ✓ | ✓ | ✓ | ✓ | Resprouter (C) |
| davitrif | *Daviesia triflora* | Fabaceae | ✓ | ✓ | ✓ |  | Resprouter (NC) * |
| desmflex | *Desmocladus flexuosus* | Restionaceae | ✓ | ✓ | ✓ | ✓ | Resprouter (C) |
| ehrhcaly | *Ehrharta calycina* | Poaceae | ✓ | ✓ | ✓ |  | Invasive perennial |
| ehrhlong | *Ehrharta longiflora* | Poaceae | ✓ | ✓ |  |  | Invasive annual |
| eremfimb | *Eremaea fimbriata* | Myrtaceae |  |  | ✓ |  | Resprouter (NC) * |
| erempauc | *Eremaea pauciflora* | Myrtaceae | ✓ | ✓ | ✓ | ✓ | Resprouter (NC) * |
| eucatodt | *Eucalyptus todtiana* | Myrtaceae | ✓ |  |  | ✓ | Resprouter (NC) * |
| gastcapi | *Gastrolobium capitatum* | Fabaceae | ✓ |  | ✓ |  | Resprouter (NC) * |
| gladcary | *Gladiolus caryophyllaceus* | Iridaceae | ✓ | ✓ | ✓ | ✓ | Invasive perennial |
| gomptome | *Gompholobium tomentosum* | Fabaceae | ✓ | ✓ | ✓ | ✓ | Obligate seeder * |
| gonopith | *Gonocarpus pithyoides* | Haloragaceae | ✓ | ✓ | ✓ | ✓ | Resprouter (C) |
| haemlaxu | *Haemodorum laxum* | Haemodoraceae |  |  | ✓ |  | Resprouter (C) * |
| haemsimp | *Haemodorum simplex* | Haemodoraceae |  |  | ✓ |  | Resprouter (C) * |
| haemspic | *Haemodorum spicatum* | Haemodoraceae |  |  | ✓ |  | Resprouter (C) * |
| helipusi | *Heliophila pusilla* | Brassicaceae |  |  | ✓ |  | Invasive annual |
| hensturb | *Hensmania turbinata* | Hemerocallidaceae | ✓ |  |  |  | Resprouter (C) |
| hibbaure | *Hibbertia aurea* | Dilleniaceae | ✓ | ✓ | ✓ |  | Resprouter (NC) |
| hibbhueg | *Hibbertia huegelii* | Dilleniaceae |  |  | ✓ |  | Resprouter (NC) |
| hibbhype | *Hibbertia hypericoides* | Dilleniaceae | ✓ | ✓ | ✓ | ✓ | Resprouter (NC) |
| hibbsubv | *Hibbertia subvaginata* | Dilleniaceae | ✓ | ✓ | ✓ | ✓ | Obligate seeder |
| homahoma | *Homalosciadium homalocarpum* | Apiaceae | | ✓ | ✓ | ✓ | Native annual |
| hovepung | *Hovea pungens* | Fabaceae | ✓ |  | ✓ |  | Obligate seeder * |
| hyalcotu | *Hyalosperma cotula* | Asteraceae | ✓ | ✓ | ✓ |  | Native annual |
| hypoangu | *Hypocalymma angustifolium* | Myrtaceae | ✓ | ✓ | ✓ |  | Resprouter (NC) |
| hypoglab | *Hypochaeris glabra* | Asteraceae | ✓ | ✓ | ✓ | ✓ | Invasive annual |
| hypolrobus | *Hypolaena robusta* | Restionaceae | |  | ✓ |  | Resprouter (NC) |
| hyporobu | *Hypocalymma robustum* | Myrtaceae | |  | ✓ |  | Obligate seeder |
| isolmarg | *Isolepis marginata* | Cyperaceae |  | ✓ | ✓ | ✓ | Native annual |
| isotcune | *Isotropis cuneifolia* | Fabaceae |  |  | ✓ | ✓ | Resprouter (C) * |
| jackflor | *Jacksonia floribunda* | Fabaceae | ✓ | ✓ | ✓ | ✓ | Resprouter (NC) * |
| jackfurc | *Jacksonia furcellata* | Fabaceae | ✓ | ✓ | ✓ | ✓ | Resprouter (NC) * |
| kunzglab | *Kunzea glabrescens* | Myrtaceae | ✓ | ✓ | ✓ |  | Obligate seeder * |
| lagehueg | *Lagenphora huegelii* | Asteraceae |  |  | ✓ |  | Native annual |
| laxmramo | *Laxmannia ramosa* | Asparagaceae |  | ✓ | ✓ | ✓ | Obligate seeder |
| laxmsqua | *Laxmannia squarrosa* | Asparagaceae | ✓ | ✓ | ✓ |  | Obligate seeder |
| lechflor | *Lechenaultia floribunda* | Goodeniaceae | ✓ | ✓ | ✓ | ✓ | Resprouter (C) |
| lepidosperma | *Lepidosperma sp.* | Cyperaceae | ✓ |  |  |  | Resprouter (C) |
| lepisqua | *Lepidosperma squamatum* | Cyperaceae |  |  | ✓ |  | Resprouter (C) |
| leptempe | *Leptomeria empetriformis* | Santalaceae |  |  | ✓ |  | Obligate seeder |
| leuccono | *Leucopogon conostephioides* | Ericaceae | ✓ |  | ✓ | ✓ | Obligate seeder |
| leucgrac | *Leucopogon gracillimus* | Ericaceae |  |  | ✓ |  | Obligate seeder |
| leucinsu | *Leucopogon insularis* | Ericaceae |  |  | ✓ |  | Obligate seeder |
| leucpoly | *Leucopogon polymorphus* | Ericaceae | ✓ | ✓ | ✓ | ✓ | Resprouter (NC) |
| leucprop | *Leucopogon propinquus* | Ericaceae | ✓ |  | ✓ |  | Resprouter (NC) |
| leucrace | *Leucopogon racemulosus* | Ericaceae |  |  | ✓ |  | Obligate seeder |
| levestip | *Levenhookia stipitata* | Stylidiaceae | ✓ | ✓ | ✓ |  | Native annual |
| lobetenu | *Lobelia tenuior* | Campanulaceae |  | ✓ |  | ✓ | Native annual |
| lomacaes | *Lomandra caespitosa* | Asparagaceae | ✓ |  | ✓ | ✓ | Resprouter (C) |
| lomaherm | *Lomandra hermaphrodita* | Asparagaceae |  |  | ✓ | ✓ | Resprouter (C) |
| lomamicr | *Lomandra micrantha* | Asparagaceae |  |  | ✓ |  | Resprouter (C) |
| lomaprei | *Lomandra preissii* | Asparagaceae |  |  | ✓ |  | Resprouter (C) |
| lomasuav | *Lomandra suaveolens* | Asparagaceae |  |  | ✓ | ✓ | Resprouter (C) |
| lygibarb | *Lyginia imberbis* | Anarthriaceae | ✓ | ✓ | ✓ | ✓ | Resprouter (C) |
| lygiimbe | *Lysimachia arvensis* | Primulaceae |  |  | ✓ | ✓ | Resprouter (C) |
| lysicili | *Lysinema ciliatum* | Ericaceae | ✓ |  |  |  | Obligate seeder |
| macaaust | *Macarthuria australis* | Molluginaceae |  |  | ✓ |  | Resprouter (C) |
| macrfras | *Macrozamia fraseri* | Zamiaceae |  |  | ✓ |  | Resprouter (NC) * |
| melaseri | *Melaleuca seriata* | Myrtaceae | ✓ | ✓ |  |  | Resprouter (NC) * |
| melasyst | *Melaleuca systena* | Myrtaceae |  |  | ✓ |  | Resprouter (NC) * |
| melatric | *Melaleuca trichophylla* | Myrtaceae | ✓ | ✓ | ✓ | ✓ | Resprouter (NC) * |
| mesopseu | *Mesomelaena pseudostygia* | Cyperaceae | ✓ |  |  |  | Resprouter (C) |
| micrstip | *Microlaena stipoides* | Poaceae |  |  | ✓ |  | Obligate seeder |
| milltenu | *Millotia tenuifolia* | Asteraceae |  | ✓ | ✓ |  | Native annual |
| neuralop | *Neurachne alopecuroidea* | Poaceae |  |  | ✓ |  | Resprouter (C) |
| nuytflor | *Nuytsia floribunda* | Loranthaceae |  |  | ✓ |  | Resprouter (C) * |
| opervagi | *Opercularia vaginata* | Rubiaceae | ✓ |  | ✓ |  | Resprouter (C) |
| pateocci | *Patersonia occidentalis* | Iridaceae | ✓ | ✓ | ✓ |  | Resprouter (C) |
| pelacapi | *Pelargonium capitatum* | Geraniaceae | ✓ | ✓ |  |  |  |
| pentairo | *Pentameris airoides* | Poaceae |  | ✓ |  | ✓ | Invasive annual * |
| perssacc | *Persoonia saccata* | Proteaceae |  |  | ✓ |  | Resprouter (NC) |
| petrdubi | *Petrorhagia dubia* | Caryophyllaceae | ✓ |  |  |  | Invasive annual * |
| petrline | *Petrophile linearis* | Proteaceae | ✓ | ✓ | ✓ | ✓ | Resprouter (NC) |
| philspic | *Philotheca spicata* | Rutaceae | ✓ |  | ✓ | ✓ | Resprouter (NC) |
| phlecili | *Phlebocarya ciliata* | Haemodoraceae |  |  | ✓ |  | Resprouter (C) |
| phylpara | *Phyllangium paradoxum* | Loganiaceae | ✓ | ✓ | ✓ | ✓ | Native annual |
| podoangu | *Podotheca angustifolia* | Asteraceae |  | ✓ | ✓ |  | Native annual |
| podochry | *Podotheca chrysantha* | Asteraceae |  |  | ✓ |  | Native annual |
| podognap | *Podotheca gnaphalioides* | Asteraceae | ✓ | ✓ | ✓ |  | Native annual |
| poramicr | *Poranthera microphylla* | Phyllanthaceae |  |  | ✓ | ✓ | Native annual |
| quinurvi | *Quinetia urvillei* | Asteraceae | ✓ | ✓ |  |  | Native annual |
| regeinop | *Regelia inops* | Myrtaceae |  |  | ✓ | ✓ | Resprouter (NC) |
| rhodchlo | *Rhodanthe chlorocephala* | Asteraceae |  | ✓ | ✓ |  | Native annual |
| rhodcitr | *Rhodanthe citrina* | Asteraceae |  |  | ✓ |  | Native annual |
| romeacet | *Acetosella vulgaris* | Polygonaceae |  |  |  | ✓ | Invasive perennial |
| romurose | *Romulea rosea* | Iridaceae | ✓ | ✓ | ✓ |  | Invasive perennial |
| rytiocci | *Rytidosperma occidentale* | Poaceae |  |  | ✓ |  | Obligate seeder |
| scaerepe | *Scaevola repens* | Goodeniaceae | ✓ | ✓ |  |  | Resprouter (NC) |
| schocurv | *Schoenus curvifolius* | Cyperaceae | ✓ | ✓ | ✓ | ✓ | Resprouter (C) |
| schoinvo | *Scholtzia involucrata* | Myrtaceae | ✓ | ✓ | ✓ | ✓ | Resprouter (NC) |
| scholaxi | *Scholtzia laxiflora* | Myrtaceae |  |  | ✓ | ✓ | Resprouter (NC) |
| schopedi | *Schoenus pedicellatus* | Cyperaceae |  |  | ✓ |  | Resprouter (C) |
| soncaspe | *Sonchus asper* | Asteraceae |  | ✓ |  |  | Invasive annual |
| soncoler | *Sonchus oleraceus* | Asteraceae | ✓ | ✓ | ✓ | ✓ | Invasive annual |
| sowelaxi | *Sowerbaea laxiflora* | Asparagaceae |  |  | ✓ | ✓ | Resprouter (C) |
| stacmono | *Stackhousia monogyna* | Celastraceae |  |  | ✓ |  | Obligate seeder |
| stirlati | *Stirlingia latifolia* | Proteaceae | ✓ | ✓ | ✓ | ✓ | Resprouter (NC) |
| styladpr | *Stylidium adpressum* | Stylidiaceae |  |  | ✓ |  | Resprouter (C) |
| stylbrun | *Stylidium brunonianum* | Stylidiaceae | ✓ | ✓ | ✓ | ✓ | Resprouter (C) |
| stylcros | *Stylidium crossocephalum* | Stylidiaceae | ✓ |  |  | ✓ | Obligate seeder |
| styldiur | *Stylidium diuroides* | Stylidiaceae | ✓ | ✓ | ✓ | ✓ | Obligate seeder |
| stylneur | *Stylidium neurophyllum* | Stylidiaceae | ✓ |  | ✓ |  | Resprouter (C) |
| stylpili | *Stylidium piliferum* | Stylidiaceae | ✓ | ✓ | ✓ | ✓ | Obligate seeder |
| stylrepe | *Stylidium repens* | Stylidiaceae | ✓ | ✓ | ✓ | ✓ | Obligate seeder |
| stylrigi | *Stylidium rigidulum* | Stylidiaceae | ✓ | ✓ | ✓ | ✓ | Obligate seeder |
| thysarbu | *Thysanotus arbuscula* | Asparagaceae | ✓ |  | ✓ |  | Resprouter (C) |
| thysaren | *Thysanotus arenarius* | Asparagaceae | ✓ |  |  |  | Resprouter (C) |
| thysmang | *Thysanotus manglesianus* | Asparagaceae |  | ✓ | ✓ |  | Resprouter (C) |
| thyspate | *Thysanotus patersonii* | Asparagaceae |  |  |  | ✓ | Obligate seeder |
| thysspar | *Thysanotus sparteus* | Asparagaceae |  |  | ✓ |  | Resprouter (C) |
| thysthyr | *Thysanotus thyrsoideus* | Asparagaceae |  |  | ✓ |  | Resprouter (C) |
| tracpilo | *Trachymene pilosa* | Araliaceae | ✓ | ✓ | ✓ | ✓ | Native annual |
| tricelat | *Tricoryne elatior* | Hemerocallidaceae |  |  | ✓ | ✓ | Resprouter (C) |
| trictene | *Tricoryne tenella* | Hemerocallidaceae |  |  | ✓ |  | Resprouter (C) |
| urospicr | *Urospermum picroides* | Asteraceae |  | ✓ |  |  | Invasive annual |
| ursianth | *Ursinia anthemoides* | Asteraceae | ✓ | ✓ | ✓ | ✓ | Invasive annual * |
| vertnite | *Verticordia nitens* | Myrtaceae | ✓ | ✓ | ✓ | ✓ | Obligate seeder |
| vulpmura | *Vulpia muralis* | Poaceae |  |  | ✓ |  | Invasive annual * |
| vulpmyur | *Vulpia myuros* | Poaceae | ✓ | ✓ | ✓ |  | Invasive annual * |
| wahlcape | *Wahlenbergia capensis* | Campanulaceae | ✓ | ✓ | ✓ | ✓ | Invasive annual |
| wahlgrac | *Wahlenbergia gracilenta* | Campanulaceae |  |  | ✓ |  | Native annual |
| wahlprei | *Wahlenbergia preissii* | Campanulaceae | ✓ | ✓ | ✓ | ✓ | Native annual |
| waitsuav | *Waitzia suaveolens* | Asteraceae |  | ✓ | ✓ |  | Native annual |
| xanthueg | *Xanthosia huegelii* | Apiaceae | ✓ | ✓ | ✓ | ✓ | Obligate seeder |
| xantprei | *Xanthorrhoea preissii* | Xanthorrhoeaceae |  |  | ✓ |  | Resprouter (NC) * |


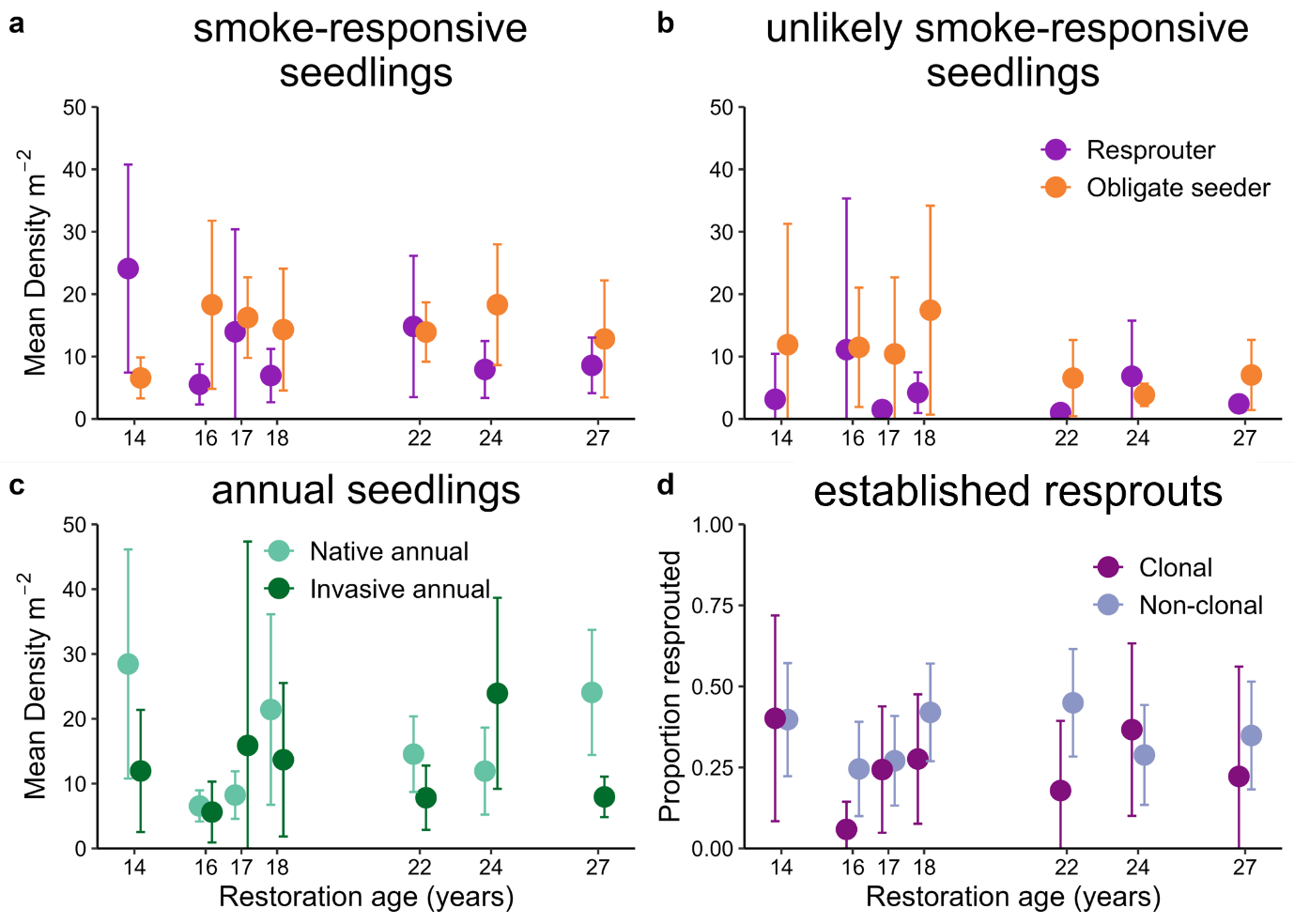


**Figure S1:** Five-month post-fire mean±95% CI of perennial a) smoke-responsive, b) unlikely smoke-responsive seedlings, c) annual seedling density (m^2^), and d) proportion of pre-fire individuals that had resprouted across the 13-year restoration chronosequence. Y axes differ, colours in b reflect those in a. n = 2–8 0.7×0.7 m plots per point for a,b & c, n = 9–25 species point for d.

**Table S2**: Effects of potential drivers on seedling and resprouting responses at five months following fire. Bolding of text indicates a significant *P* value at = <0.05 level, × denotes an interaction, τ00 refers to individual random intercept variance explained.

| *Response* | *Predictor* | *Estimate* | *SE* | *Z value* | *P* |
| --- | --- | --- | --- | --- | --- |
| Perennial seedlings | **Intercept** | **1.377** | **0.282** | **4.884** | **<0.001** |
|  | Restoration age | -0.098 | 0.096 | -1.028 | 0.950 |
|  | Fire response [obligate seeder] | 0.163 | 0.278 | 0.586 | 0.950 |
|  | Smoke response [yes] | 0.372 | 0.310 | 1.200 | 0.950 |
|  | Fire coverage | 0.138 | 0.115 | 1.195 | 0.950 |
|  | Age × fire response [obligate seeder] | 0.283 | 0.113 | 2.508 | 0.088 |
|  | Smoke response [yes] × fire coverage | -0.225 | 0.126 | -1.788 | 0.380 |
|  | *Random effects* |  |  |  |  |
|  | τ_00 age_ | <0.001 |  |  |  |
|  | τ_00 species_ | 0.770 |  |  |  |
|  | Marginal R^2^ / Conditional R^2^ | 0.025 / 0.319 | |  |  |
| Annual seedlings | **Intercept** | **2.249** | **0.233** | **9.662** | **<0.001** |
|  | Restoration age | 0.149 | 0.153 | 0.951 | 0.951 |
|  | Status [invasive] | -0.303 | 0.331 | -0.915 | 0.951 |
|  | Fire coverage | 0.047 | 0.115 | 0.409 | 0.951 |
|  | Status[invasive] × age | 0.243 | 0.195 | 1.247 | 0.097 |
|  | Status[invasive] × fire coverage | -0.386 | 0.187 | -2.058 | 0.323 |
|  | *Random effects* |  |  |  |  |
|  | τ_00 age_ | 0.071 |  |  |  |
|  | τ_00 species_ | 0.486 |  |  |  |
|  | Marginal R^2^ / Conditional R^2^ | 0.021 / 0.217 | |  |  |
| Established resprouts | Intercept | -0.097 | 0.167 | -0.581 | 0.999 |
|  | Restoration age | -0.055 | 0.118 | -0.470 | 0.999 |
|  | Fire severity | 0.103 | 0.122 | 0.848 | 0.999 |
|  | Clonal [yes] | -0.515 | 0.259 | -1.987 | 0.282 |
|  | Age × clonal [yes] | 0.077 | 0.207 | 0.372 | 0.999 |
|  | Fire severity × clonal [yes] | -0.023 | 0.190 | -0.122 | 0.999 |
|  | *Random effects* |  |  |  |  |
|  | τ_00 age_ | <0.001 |  |  |  |
|  | τ_00 species_ | 0.278 |  |  |  |
|  | Marginal R^2^ / Conditional R^2^ | 0.024 / 0.072 | |  |  |


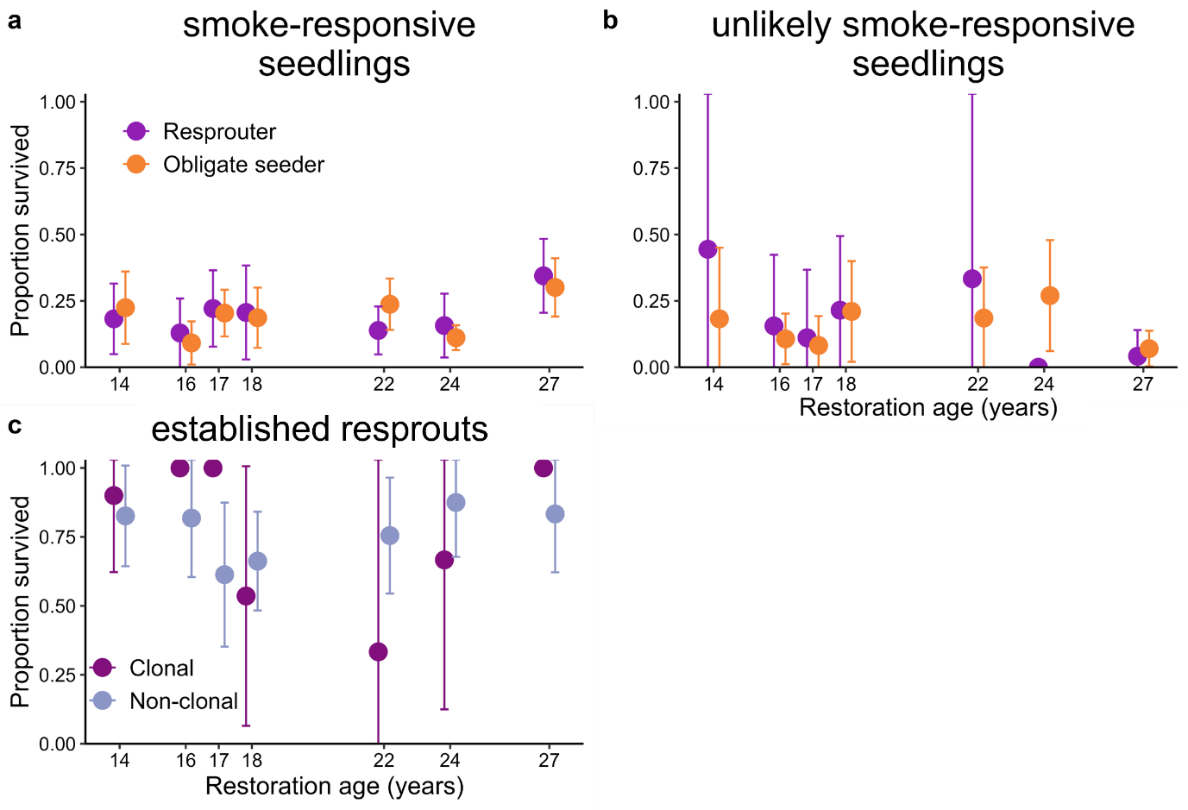
 **Figure S2: ~**17-month mean±95% CI of post-fire survival proportions of perennial a) smoke-responsive, b) unlikely smoke-responsive perennial seedlings and c) established resprouts across the 13-year restoration chronosequence. n = 3–42 species per point for a and b, d = 2–19 species per point.

**Table S3:** Effects of potential drivers of survival of perennial seedings and established resprouts. Bolding of text indicates a significant *P* value at = <0.05 level, × denotes an interaction, τ00 refers to individual random intercept variance explained.

| *Response* | *Predictor* | *Estimate* | *SE* | *Z value* | *P* |
| --- | --- | --- | --- | --- | --- |
| Perennial seedlings | **Intercept** | **-1.231** | **0.151** | **-8.163** | **<0.001** |
|  | Restoration age | -0.004 | 0.097 | -0.038 | 0.999 |
|  | Fire response [obligate seeder] | 0.103 | 0.127 | 0.809 | 0.999 |
|  | Smoke response [yes] | 0.172 | 0.142 | 1.213 | 0.999 |
|  | Compaction (10 cm) | 0.083 | 0.063 | 1.305 | 0.999 |
|  | Field capacity | 0.003 | 0.059 | 0.052 | 0.999 |
|  | Age × fire response [obligate seeder] | 0.072 | 0.114 | 0.635 | 0.999 |
|  | *Random effects* |  |  |  |  |
|  | τ_00 age_ | <0.001 |  |  |  |
|  | τ_00 species_ | 0.029 |  |  |  |
|  | Marginal R^2^ / Conditional R^2^ | 0.012 / 0.015 | |  |  |
| Established resprouts | **Intercept** | **0.690** | **0.146** | **4.734** | **<0.001** |
|  | Restoration age | 0.034 | 0.149 | 0.22 | 0.999 |
|  | Clonal [yes] | -0.002 | 0.277 | -0.008 | 0.999 |
|  | Compaction (30 cm) | 0.089 | 0.135 | 0.657 | 0.999 |
|  | Field capacity | -0.037 | 0.135 | -0.275 | 0.999 |
|  | Age × Clonal [yes] | -0.228 | 0.280 | -0.814 | 0.999 |
|  | *Random effects* |  |  |  |  |
|  | τ_00 age_ | <0.001 |  |  |  |
|  | τ_00 species_ | <0.001 |  |  |  |
|  | Marginal R^2^ / Conditional R^2^ | 0.012 / 0.012 | |  |  |

**Table S4**: Indicator species per age and treatment type. Species with ” are those found in either the restored or reference sites, but not both.

| Treatment | Age | Trait type | Species |
| --- | --- | --- | --- |
| Pre-fire restoration | 16 | Resprouter | *Gastrolobium capitatum* |
|  | 17 | Resprouter | *Melaleuca seriata* ” |
|  | 17 | Resprouter | *Lepidosperma sp* ” |
|  | 24 | Resprouter | *Allocasuarina humilis* |
|  | 24 | Invasive annual | *Aira caryophyllea* |
|  | 27 | Resprouter | *Hensmania turbinata* ” |
|  | 27 | Resprouter | *Opercularia vaginata* |
|  | 27 | Invasive annual | [*Petrorhagia*](https://en.wikipedia.org/wiki/Petrorhagia_dubia) *dubia* |
| Post-fire restoration | 14 | Resprouter | *Scholtzia involucrata* |
|  | 17 | Invasive annual | *Avena barbata* |
|  | 18 | Resprouter | *Hypocalymma angustifolium* |
|  | 18 | Native annual | *Centrolepis glabra* |
|  | 22 | Obligate seeder | *Verticordia nitens* |
|  | 22 | Native annual | *Hyalosperma cotula* |
|  | 22 | Native annual | *Phyllagium paradoxum* |
|  | 24 | Obligate seeder | *Laxmannia squarrosa* |
|  | 24 | Invasive annual | *Ursinia anthemoides* |
|  | 24 | Invasive annual | *Pentameris airoides* |
|  | 27 | Resprouter | Gonocarpus pithyoides |
|  | 27 | Native annual | Waitzia suaveolens |
|  | 27 | Native annual | *Wahlenbergia preissii* |
|  | 27 | Native annual | *Isolepis marginata* |
|  | 27 | Native annual | *Austrostipa compressa* |
|  | 27 | Native annual | *Trachymene pilosa* |
|  | 27 | Invasive annual | *Hypochaeris glabra* |
|  | 27 | Invasive annual | *Brixa maxima* |
|  | 27 | Invasive annual | *Sonchus oleraceus* |
| Pre-fire reference | 4 | Resprouter | *Scholtzia laxiflora* ” |
|  | 49 | Resprouter | *Alexgeorgea nitens* |
|  | 49 | Resprouter | *Leucopogon propinquus* ” |
| Post-fire reference | 10 | Resprouter | *Lomandra suaveolens* |
|  | 10 | Resprouter | *Banksia menziesii* |
|  | 10 | Resprouter | *Lyginia barbata* |
|  | 22 | Resprouter | *Melaleuca trichophylla* |
|  | 22 | Resprouter | *Lyginia imberbis* |
|  | 22 | Resprouter | *Eremaea pauciflora* |
|  | 22 | Resprouter | *Burchardia congesta* |
|  | 22 | Obligate seeder | *Leucopogon conostephioides* |
|  | 22 | Obligate seeder | *Stylidium rigidulum* |
|  | 22 | Obligate seeder | *Calytrix flavescens* |
|  | 22 | Obligate seeder | *Hibbertia subvaginata* |
|  | 22 | Obligate seeder | *Stylidium repens* |
|  | 22 | Obligate seeder | Laxmannia ramosa |
|  | 22 | Obligate seeder | *Xanthosia huegelii* |
|  | 22 | Obligate seeder | *Thysanotus patersonii*” |
|  | 22 | Obligate seeder | *Stylidium diuroides* |

**References:**

Akaike, H. 1973. "Information theory and an extension of the maximum likelihood principle." Second International Symposium on Information Theory, Budapest.

Alauddin, Mohammad, and Hong Son Nghiemb. 2010. "Do Instructional Attributes pose Multicollinearity Problems? An Empirical Exploration." *Economic Analysis and Policy* 40 (3):351-361. doi: <https://doi.org/10.1016/S0313-5926(10)50034-1>.

Barton, K. (2022) *MuMIn: Multi-Model Inference*. (R package version 1.46.0). Available at: https://CRAN.R-project.org/package=MuMIn

Baskin, C. C. & Baskin, J. M. (2014) *Seeds, Ecology, Biogeography and Evolution of Dormancy and Germination*. San Diego, California, USA: Elsevier.

Brooks, M. E., Kristensen, K., van Benthem, K. J., Magnusson, A., Berg, C. W., Nielsen, A., Skaug, H. J., Mächler, M. and Bolker, B. B. (2017) 'glmmTMB balances speed and flexibility among packages for zero-inflated generalized linear mixed modelling', *The R Journal,* 9(2), pp. 378-400.

Burnham, K.P., and D.R. Anderson. 2004. "Multimodel inference: understanding AIC and BIC in model selection." *Sociological Methods & Research* 33 (2):261–304. doi: https://doi.org/10.1177/004912410426864.

Clarke, P. J., M. J. Lawes, J. J. Midgley, B. B. Lamont, F. Ojeda, G. E. Burrows, N. J. Enright, and K. J. E. Knox. 2013. "Resprouting as a key functional trait: how buds, protection and resources drive persistence after fire." *New Phytologist* 197 (1):19-35. doi: https://doi.org/10.1111/nph.12001.

Cowan, Ebony L., Joseph B. Fontaine, Rachel J. Standish, and Ben P. Miller. 2023a. "Drivers of post-fire resprouting success in restored Banksia woodlands." *Austral Ecology* 48 (8):2088-2107. doi: https://doi.org/10.1111/aec.13447.

Cowan, Ebony L., Ben P. Miller, Joseph B. Fontaine, Neal J. Enright, and Rachel J. Standish. 2023b. "Soil seed bank development of smoke-responsive plant species in a 23-year restoration chronosequence and implications for resilience to fire." *Applied Vegetation Science* 26 (1):e12713. doi: https://doi.org/10.1111/avsc.12713.

Cox D. R., and Snell E. J. 1989. *Analysis of Binary Data*. 2 ed. New York: Chapman and Hall.

Enright, Neal J., Joseph B. Fontaine, Byron B. Lamont, Ben P. Miller, and Vanessa C. Westcott. 2014. "Resistance and resilience to changing climate and fire regime depend on plant functional traits." *Journal of Ecology* 102 (6):1572-1581. doi: https://doi.org/10.1111/1365-2745.12306.

Falster, Daniel, Rachael Gallagher, Elizabeth H. Wenk, Ian J. Wright, Dony Indiarto, Samuel C. Andrew, Caitlan Baxter, James Lawson, Stuart Allen, Anne Fuchs, Anna Monro, Fonti Kar, Mark A. Adams, Collin W. Ahrens, Matthew Alfonzetti, Tara Angevin, Deborah M. G. Apgaua, Stefan Arndt, Owen K. Atkin, Joe Atkinson, Tony Auld, Andrew Baker, Maria von Balthazar, Anthony Bean, Chris J. Blackman, Keith Bloomfield, David M. J. S. Bowman, Jason Bragg, Timothy J. Brodribb, Genevieve Buckton, Geoff Burrows, Elizabeth Caldwell, James Camac, Raymond Carpenter, Jane A. Catford, Gregory R. Cawthray, Lucas A. Cernusak, Gregory Chandler, Alex R. Chapman, David Cheal, Alexander W. Cheesman, Si-Chong Chen, Brendan Choat, Brook Clinton, Peta L. Clode, Helen Coleman, William K. Cornwell, Meredith Cosgrove, Michael Crisp, Erika Cross, Kristine Y. Crous, Saul Cunningham, Timothy Curran, Ellen Curtis, Matthew I. Daws, Jane L. DeGabriel, Matthew D. Denton, Ning Dong, Pengzhen Du, Honglang Duan, David H. Duncan, Richard P. Duncan, Marco Duretto, John M. Dwyer, Cheryl Edwards, Manuel Esperon-Rodriguez, John R. Evans, Susan E. Everingham, Claire Farrell, Jennifer Firn, Carlos Roberto Fonseca, Ben J. French, Doug Frood, Jennifer L. Funk, Sonya R. Geange, Oula Ghannoum, Sean M. Gleason, Carl R.

Gosper, Emma Gray, Philip K. Groom, Saskia Grootemaat, Caroline Gross, Greg Guerin, Lydia Guja, Amy K. Hahs, Matthew Tom Harrison, Patrick E. Hayes, Martin Henery, Dieter Hochuli, Jocelyn Howell, Guomin Huang, Lesley Hughes, John Huisman, Jugoslav Ilic, Ashika Jagdish, Daniel Jin, Gregory Jordan, Enrique Jurado, John Kanowski, Sabine Kasel, Jürgen Kellermann, Belinda Kenny, Michele Kohout, Robert M. Kooyman, Martyna M. Kotowska, Hao Ran Lai, Etienne Laliberté, Hans Lambers, Byron B. Lamont, Robert Lanfear, Frank van Langevelde, Daniel C. Laughlin, Bree-Anne Laugier-Kitchener, Susan Laurance, Caroline E. R. Lehmann, Andrea Leigh, Michelle R. Leishman, Tanja Lenz, Brendan Lepschi, James D. Lewis, Felix Lim, Udayangani Liu, Janice Lord, Christopher H. Lusk, Cate Macinnis-Ng, Hannah McPherson, Susana Magallón, Anthony Manea, Andrea López-Martinez, Margaret Mayfield, James K. McCarthy, Trevor Meers, Marlien van der Merwe, Daniel J. Metcalfe, Per Milberg, Karel Mokany, Angela T. Moles, Ben D. Moore, Nicholas Moore, John W. Morgan, William Morris, Annette Muir, Samantha Munroe, Áine Nicholson, Dean Nicolle, Adrienne B. Nicotra, Ülo Niinemets, Tom North, Andrew O’Reilly-Nugent, Odhran S. O’Sullivan, Brad Oberle, Yusuke Onoda, Mark K. J. Ooi, Colin P. Osborne, Grazyna Paczkowska, Burak Pekin, Caio Guilherme Pereira, Catherine Pickering, Melinda Pickup, Laura J. Pollock, Pieter Poot, Jeff R. Powell, Sally A. Power, Iain Colin Prentice, Lynda Prior, Suzanne M. Prober, Jennifer Read, Victoria Reynolds, Anna E. Richards, Ben Richardson, Michael L. Roderick, Julieta A. Rosell, Maurizio Rossetto, Barbara Rye, Paul D. Rymer, Michael A. Sams, Gordon Sanson, Hervé Sauquet, Susanne Schmidt, Jürg Schönenberger, Ernst-Detlef Schulze, Kerrie Sendall, Steve Sinclair, Benjamin Smith, Renee Smith, Fiona Soper, Ben Sparrow, Rachel J. Standish, Timothy L. Staples, Ruby Stephens, Christopher Szota, Guy Taseski, Elizabeth Tasker, Freya Thomas, David T. Tissue, Mark G. Tjoelker, David Yue Phin Tng, Félix de Tombeur, Kyle Tomlinson, Neil C. Turner, Erik J. Veneklaas, Susanna Venn, Peter Vesk, Carolyn Vlasveld, Maria S. Vorontsova, Charles A. Warren, Nigel Warwick, Lasantha K. Weerasinghe, Jessie Wells, Mark Westoby, Matthew White, Nicholas S. G. Williams, Jarrah Wills, Peter G. Wilson, Colin Yates, Amy E. Zanne, Graham Zemunik, and Kasia Ziemińska. 2021. "AusTraits, a curated plant trait database for the Australian flora." *Scientific Data* 8 (1):254. doi: https://doi.org/10.1038/s41597-021-01006-6.

Geissinger, Emilie A., Celyn L. L. Khoo, Isabella C. Richmond, Sally J. M. Faulkner, and David C. Schneider. 2022. "A case for beta regression in the natural sciences." *Ecosphere* 13 (2):e3940. doi: https://doi.org/10.1002/ecs2.3940.

Holm S. (1979) 'A simple sequentially rejective multiple test procedure', *Scandinavian Journal of Statistics,* 6, pp. 65-70.

Lüdecke, D., Ben-Shachar, M. S., Patil, I., Waggoner, P. and Makowski, D. (2021) 'Performance: an R package for assessment, comparison and testing of statistical models', *Journal of Open Source Software,* 6(60), pp. 3139.

Mangiafico S. S. (2023) *rcompanion: Functions to Support Extension Education Program Evaluation* (R package version 2.4.34). Available at: https://CRAN.R-project.org/package=rcompanion

McCullagh, P. and Nelder, J. A. (1989) *Generalized Linear Models.* 2nd edn. London, United Kingdom: Chapman and Hall

Nakagawa, Shinichi, Paul C. D. Johnson, and Holger Schielzeth. 2017. "The coefficient of determination R2 and intra-class correlation coefficient from generalized linear mixed-effects models revisited and expanded." *Journal of The Royal Society Interface* 14 (134):20170213. doi: <https://doi.org/10.1098/rsif.2017.0213>.

Offord, C. A. & Meagher, P. F. (2009) *Plant Germplasm Conservation in Australia: Strategies and Guidelines for Developing, Managing and Utilising Ex Situ Collections, full rev. edn.,* Canberra, Australia: Australian Network for Plant Conservation. Available at: <https://www.anpc.asn.au/wp-content/uploads/2021/02/Plant-Germplasm-Conservation-InAustralia-online.pdf> (Accessed: 16 March 2021)

Ospina, Raydonal, and Silvia L. P. Ferrari. 2010. "Inflated beta distributions." *Statistical Papers* 51 (1):111-126. doi: https://doi.org/10.1007/s00362-008-0125-4.

Pausas, Juli G., and Jon E. Keeley. 2014. "Evolutionary ecology of resprouting and seeding in fire-prone ecosystems." *New Phytologist* 204 (1):55-65. doi: https://doi.org/10.1111/nph.12921.

Pausas, Juli G., Byron B. Lamont, Susana Paula, Beatriz Appezzato-da-Glória, and Alessandra Fidelis. 2018. "Unearthing belowground bud banks in fire-prone ecosystems." *New Phytologist* 217 (4):1435-1448. doi: <https://doi.org/10.1111/nph.14982>.

R Core Team (2020) *R: A language and environment for statistical computing.* Vienna, Austria: R Foundation for Statistical Computing. Available at: https://www.R-project.org/

Schielzeth, Holger, Niels J. Dingemanse, Shinichi Nakagawa, David F. Westneat, Hassen Allegue, Céline Teplitsky, Denis Réale, Ned A. Dochtermann, László Zsolt Garamszegi, and Yimen G. Araya-Ajoy. 2020. "Robustness of linear mixed-effects models to violations of distributional assumptions." *Methods in Ecology and Evolution* 11 (9):1141-1152. doi: https://doi.org/10.1111/2041-210X.13434.

Schielzeth H. 2010. "Simple means to improve the interpretability of regression coefficients." *Methods in Ecology and Evolution* 1 (2):103-113. doi: <https://doi.org/10.1111/j.2041-210X.2010.00012.x>.

Stevens, J. C., Rokich, D. P., Newton, V. J., Barrett, R. L. and Dixon, K. W. (2016) *Banksia Woodlands - A Restoration Guide for the Swan Coastal Plain.* Nedlands, Western Australia, Australia: University of Western Australia Press.

Sweedman, B. S. & Merritt, D. J. (eds) (2006) *Australian Seeds: A Guide to their Collection, Identification and Biology*. Victoria, Australia: CSIRO Publishing.

Tsakalos J.L., Riviera F., Veneklaas E.J., Dobrowlski M.P., Mucina L. (2020) Data from: Trait-based formal definition of plant functional types and functional communities in the multi-species and multi-traits context. https://doi.org/10.1016/j.ecocom.2019.100787

Veber et al., (2019) Unpublished database.

Western Australian Herbarium (1998–). Florabase—the Western Australian Flora. Department of Biodiversity, Conservation and Attractions. <https://florabase.dpaw.wa.gov.au/>

Wilson, B.A., J. Kuehs, and L.E. Valentine. 2010. Guidelines for developing ecological burning regimes for the Gnangara groundwater system. edited by Department of Environment and Conservation. Perth, Western Australia.

Western Australia: Department of Environment and Conservation. <https://researchportal.murdoch.edu.au/esploro/outputs/report/Guidelines-for-developing-ecological-burning-regimes/99100554124600789> (Accessed: 27th January 2022).

Zuur A.F., Ieno E.N., Walker N.J., Saveliev A.A., and Smith G.M. 2009. *Mixed effect models and extensions in Ecology with R*. New York: Springer.
